# Crocetin antagonizes parthanatos in ischemic stroke via inhibiting NOX2 and preserving mitochondrial hexokinase-I

**DOI:** 10.1038/s41419-023-05581-x

**Published:** 2023-01-21

**Authors:** Hao Wu, Ying Li, Qian Zhang, Hanxun Wang, Wenyu Xiu, Pu Xu, Yujie Deng, Wanxu Huang, Dan Ohtan Wang

**Affiliations:** 1 Wuya College of Innovation, Shenyang Pharmaceutical University, Shenyang, 110016 China; 2 College of Pharmaceutical Engineering, Shenyang Pharmaceutical University, Shenyang, 110016 China; 3 Department of Pharmacology, Shenyang Pharmaceutical University, Shenyang, 110016 China; 4 Guangzhou National Laboratory, Guangzhou, Guangdong 510530 China; 5 The Fifth Affiliated Hospital of Guangzhou Medical University, Guangzhou, Guangdong 510700 China; 6 State Key Laboratory of New-tech for Chinese Medicine Pharmaceutical Process, Lianyungang, Jiangsu 222001 China; 7 Center for Biosystems Dynamics Research, RIKEN, 2-2-3 Minatojima-Minamimachi, Chuo-Ku, Kobe, Hyogo 650-0047 Japan; 8 Graduate School of Biostudies, Kyoto University, Yoshida Hon-Machi, Kyoto, 606-8501 Japan

**Keywords:** Cell death in the nervous system, Ubiquitylated proteins, Target identification, PolyADP-ribosylation, Protein translocation

## Abstract

Parthanatos is one of the major pathways of programmed cell death in ischemic stroke characterized by DNA damage, poly (ADP-ribose) polymerases (PARP) activation, and poly (ADP-ribose) (PAR) formation. Here we demonstrate that crocetin, a natural potent antioxidant compound from *Crocus sativus*, antagonizes parthanatos in ischemic stroke. We reveal that mechanistically, crocetin inhibits NADPH oxidase 2 (NOX2) activation to reduce reactive oxygen species (ROS) and PAR production at the early stage of parthanatos. Meanwhile we demonstrate that PARylated hexokinase-I (HK-I) is a novel substrate of E3 ligase RNF146 and that crocetin interacts with HK-I to suppress RNF146-mediated HK-I degradation at the later stage of parthanatos, preventing mitochondrial dysfunction and DNA damage that ultimately trigger the irreversible cell death. Our study supports further development of crocetin as a potential drug candidate for preventing and/or treating ischemic stroke.

## Introduction

Stroke constitutes the second leading cause of death and the leading cause of disability worldwide [1]. Among all stroke cases, 70% are ischemic stroke (IS) [2]. Despite significant progress in understanding of the pathological basis of IS-caused brain damage, clinical management remains limited, and the standard interventions are to restore bloodstream by pharmacological thrombolysis or mechanical thrombectomy. Only 11% of ischemic stroke patients are currently receiving intervention in the form of recombinant tissue plasminogen activator (r-tPA), and less than half of these patients show significant improvement [3]. Therefore, a formidable challenge remains to identify tractable pharmaceutical targets as well as effective and safe drugs that can be used in clinic sectors.

Parthanatos is a major type of programmed cell death in ischemic stroke [4, 5], which is dependent on the activation of poly (ADP-ribose) polymerases (PARP) and the formation of poly (ADP-ribose) (PAR) [6]. The initial stage involves DNA damage-induced PARP overactivation which catalyzes PAR and results in its accumulation. Parthanatos is reversible until the final stage which involves loss of mitochondrial membrane potential and translocation of apoptosis inducing factor (AIF) from mitochondria to nucleus and subsequent chromatin degradation [7, 8]. Oxidative stress plays a key role in cell parthanatos, which could be induced by oxygen glucose deprivation (OGD), DNA-alkylating agent N-methyl-N-nitro-N-nitrosoguanidine (MNNG) or excitotoxicity[9–11]. The NADPH oxidase (NOX) family is responsible for early-phase ROS production in MNNG-induced parthanatos [10], among which NOX2 is primarily expressed in neurons [12, 13] and is up-regulated and activated in the peri-infarct regions at early time points after stroke [14–18].

Mitochondrial damage also plays a critical role in parthanatos, and the binding of hexokinases (HK) to mitochondria has been demonstrated to be protective of mitochondrial function during ischemic injury by preventing the opening of the mitochondrial permeability transition pore [19]. It has been reported in parthanatos that PARP-1 activation leads to NAD^+^-independent glycolytic and bioenergetic failure that is caused by inhibition of HK activity by PARylation [20, 21].

In the current study, we demonstrate that crocetin, a natural product from *Crocus sativus*, reduces damage during ischemic stroke by antagonizing neuronal parthanatos. We also reveal the underlying molecular mechanisms and provide evidence to suggest that crocetin is a potential drug candidate against ischemic stroke.

## Results

### Crocin ameliorates neuronal parthanatos in pMCAO rats

**Fig. 1:**
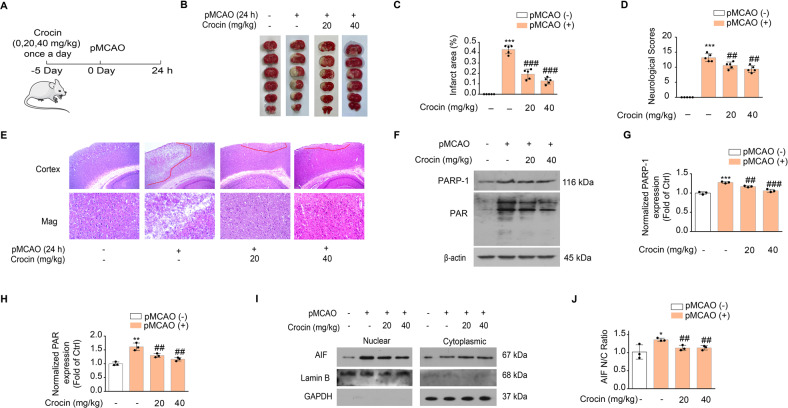
Alleviation of brain injury in pMCAO rats after crocin pre-administration. **A** Scheme diagram of the experiment. Rats were pre-dosed with crocin (0, 20, and 40 mg/kg, p.o) once a day for 5 days before pMCAO for 24 h (*n* = 5 per group). **B** TTC staining of brains slices collected at 24 h after onset of IS. **C** Quantification of cerebral infarct areas assessed by TTC staining. **D** Average modified neurological severity scores (mNSS). **E** Histopathological examination was performed by H&E staining. **F**–**H** Western blot (**F**) and group quantification of PARP-1 (**G**) and PAR (**H**) from three independent experiments. **I** AIF translocation was assessed by western blot. **J** Group quantification of gray intensity in (**I**) from three independent experiments. Significance was determined by one-way ANOVA. *p < 0.05, **p < 0.01, ***p < 0.001, vs control group; ^##^*p* < 0.01, ^###^*p* < 0.001, vs pMCAO group.

We first set up a rat ischemic stroke model using the permanent middle cerebral artery occlusion (pMCAO) to test the potential neuroprotective effect of crocetin. Since crocin is metabolized into crocetin which can directly cross the brain–blood barrier (BBB) in vivo [22], we orally administrated male SD rats with crocin (0, 20, 40 mg/kg) daily for five days prior to pMCAO (Fig. 1A). Neurological functional assessment using mNSS and size of infarct 24 h after pMCAO were used to evaluate brain damage and disability. As indicated in Fig. 1B–D, pMCAO induced massive tissue damage in the unilateral side of the brain (43.11 ± 0.04 %), while administration of crocin (20, 40 mg/kg) not only reduced brain infarct area (19.31 ± 0.04% 20 mg/kg, 12.91 ± 0.03% 40 mg/kg) but also ameliorated neurological deficit (13.20 ± 1.30 vs 10.60 ± 1.14 20 mg/kg, *p* < 0.01; 13.20 ± 1.30 vs 9.40 ± 1.14 40 mg/kg, *p* < 0.01), in a dose-dependent manner. Consistently, the histological sections revealed that pMCAO induced disbandment of cortical structures in the ischemic brain areas and extensive cell death, which were significantly alleviated by crocin in dose-dependent manner (Fig. 1E).

To test whether the protective effect of crocetin is against parthanatos, we probed the expression levels of PARP-1 and PAR associated with pMCAO. Consistent with previous reports, pMCAO caused drastic increase of PARP-1 and PAR indicating parthanatos, which was however, significantly prevented by the prior administration of crocin (Fig. 1F–H). Furthermore, we performed nuclear–cytoplasm fractionation and tested subcellular distribution of AIF, a marker protein whose nuclear translocation marks irreversible cell death by parthanatos. Crocin administration significantly reduced nuclear/cytoplasmic ratio of AIF protein (1.36 ± 0.04 vs 1.14 ± 0.07 fold of control 20 mg/kg, *p* < 0.01; 1.36 ± 0.04 vs 1.13 ± 0.06 fold of control 40 mg/kg, *p* < 0.01) after pMCAO procedure (Fig. 1I, J). These results showed reduced parthanatos during IS when animals were administrated with crocin.

### Crocetin reduces neuronal parthanatos induced by OGD or excitotoxicity

**Fig. 2:**
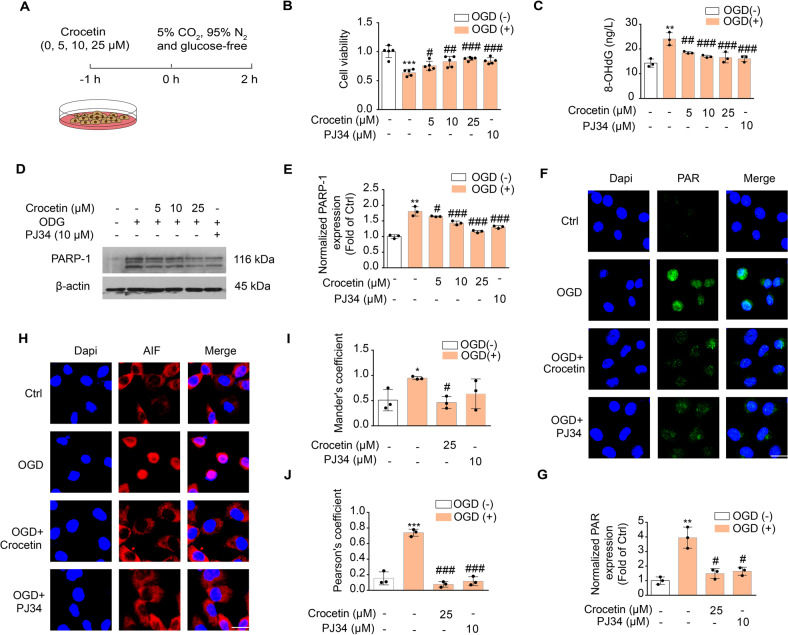
Anti-parthanatos effects of crocetin in the OGD cell model. **A** Scheme diagram of the experiment. SH-SY5Y cells were treated with indicated concentrations of crocetin or PJ34 (PARP inhibitor, 10 μM) for 1 h before 2 h-OGD (there is no crocetin or PJ34 treatment during OGD). **B** Cell viability of SH-SY5Y at the end of 2 h-OGD determined with MTT assay. **C** OGD-induced 8-OHdG in SH-SY5Y cells at the end of 2 h-OGD. **D** OGD-induced PARP-1 expression SH-SY5Y cells upon crocetin or PJ34 pre-treatment. **E** Group quantification of (**D**) from three independent experiments. **F** Confocal images of SH-SY5Y cells immuno-stained with PAR-specific antibody (green) and DAPI (blue). Cells were pretreated with crocetin (25 μM) for 1 h before 2 h-OGD. Scale bar: 10 μm. **G** Group quantification of fluorescence intensity in (**F**). **H** Confocal images of SH-SY5Y cells immuno-stained with AIF-specific antibody (red) and DAPI (blue). Cells were pretreated with crocetin (25 μM) for 1 h before 2 h-OGD. Scale bar: 10 μm. **I** Colocalization coefficient between AIF and DAPI measured as Mander’s coefficient. **J** Pearson’s correlation coefficient between AIF and DAPI staining. Significance was determined by one-way ANOVA. *p < 0.05, **p < 0.01, ***p < 0.001, vs. control group; ^#^*p* < 0.05, ^##^*p* < 0.01, ^###^*p* < 0.001, vs. OGD group.

One hour before the start of oxygen glucose deprivation (OGD), neuron cells were incubated in 5, 10, 25 μM crocetin-containing normal medium (Fig. 2A). As expected, 2-h of OGD reduced cell viability rate to 63.96 ± 0.06%, but less cell death was observed upon crocetin pre-treatment in a dose-dependent manner (Fig. 2B). Selective PARP inhibitor PJ34 promoted neuronal survival to the similar level of crocetin pre-treatment, suggesting crocetin may effectively promote cell survival through anti-parthanatos pathway as PJ34. To further confirm, we evaluated DNA damage by 8-OHdG (Fig. 2C), PARP-1 expression (Fig. 2D, E), PAR formation (Fig. 2F, G) and AIF nuclear-translocation (Fig. 2H–J). All measures consistently demonstrated that like PJ34, crocetin pre-incubation effectively inhibited parthanatos-associated cellular events and OGD-induced cell death. We further assessed the preventive effect of crocetin on secondary neuron damage after OGD (Fig. S1A). As shown in Fig. S1B–Q, parthanatos participated in the secondary cell damage characterized by 8-OHdG generation, PARP-1 activation, PAR formation and AIF nuclear-translocation in 4 or 24 h after OGD, which were reversed by PJ34. Conspicuously, crocetin reduced neuron death and parthanatos-linked biomarkers in a concentration-dependent manner not only in 4 h after OGD (Fig. S1B–I) but also 24 h after OGD (Fig. S1J–Q).

**Fig. 3:**
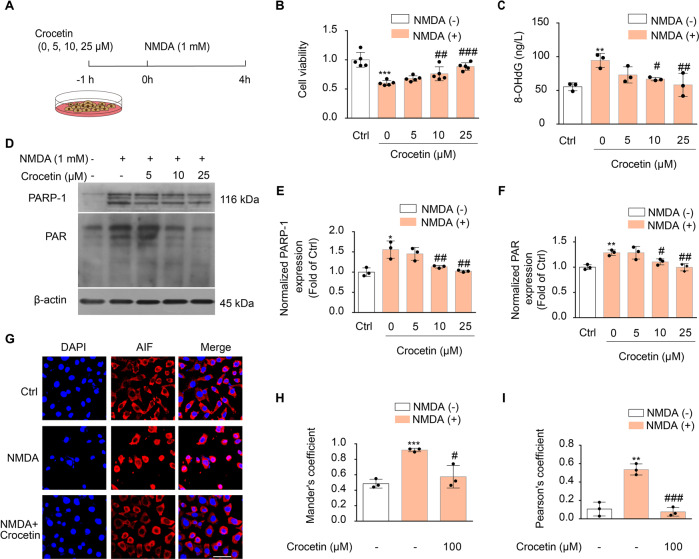
Crocetin protected neurons against excitotoxicity-induced parthanatos. **A** Scheme diagram of the experiment. Cells were pre-incubated with indicated concentrations of crocetin for 1 h before NMDA (1 mM) stimulation for 4 h. **B** Cell viability of SH-SY5Y cells determined with MTT assay. **C** NMDA-induced 8-OHdG in SH-SY5Y cells. **D** NMDA-induced PARP-1 and PAR expression in SH-SY5Y cells. **E** Group quantification of PARP-1 in (**D**) from three independent experiments. **F** Group quantification of PAR in (**D**) from three independent experiments. **G** Confocal images of SH-SY5Y cells immuno-stained with AIF-specific antibody (red) and DAPI (blue). Cells were pretreated with crocetin (25 μM) for 1 h before NMDA (1 mM) stimulation for 4 h. Scale bar: 20 μm. **H** Colocalization coefficient between AIF and DAPI measured as Mander’s coefficient. **I** Pearson’s correlation coefficient between AIF and DAPI staining. Significance was determined by one-way ANOVA. *p < 0.05, **p < 0.01, ***p < 0.001, vs. control group; ^#^*p* < 0.05, ^##^*p* < 0.01, ^###^*p* < 0.001, vs NMDA group.

Glutamate excitotoxicity-triggered parthanatos is caused by the large influx of calcium through the N-methyl-D-aspartate (NMDA) type of glutamate receptors. We found that pre-incubation of crocetin one hour before the NMDA stimulation effectively promoted the survival of neurons in a concentration-dependent manner (Fig. 3A, B). Similar to the OGD model, crocetin effectively inhibited parthanatos-associated cellular events such as elevated expression of 8-OHdG (Fig. 3C), PARP-1 and PAR (Fig. 3D–F), and nuclear translocation of AIF (Fig. 3G–I). Thus, these findings consistently demonstrated that crocetin antagonizes parthanatos and suppress ischemic stroke-induced tissue damage upon multiple cellular insults.

### Crocetin ameliorates MNNG-induced pathanatos in both early and late phases

**Fig. 4:**
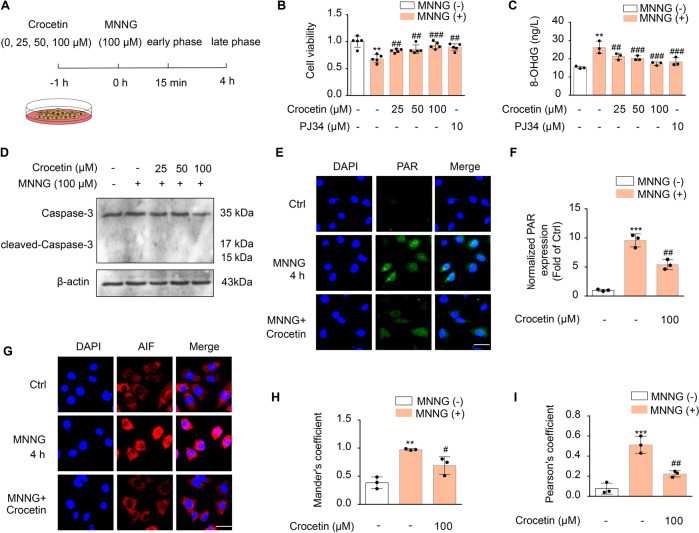
Anti-pathanatos effects of crocetin at late pathanatotic phases. **A** Scheme diagram of the experiment. Cells were incubated with indicated concentrations of crocetin for 1 h before MNNG (100 μM) stimulations for 15 min or 4 h. **B** Cell viability of SH-SY5Y cells after 4 h-MNNG determined with MTT assay. Data represent mean ± SD from five independent experiments. **C** 4 h-MNNG-induced 8-OHdG (ng/L) in SH-SY5Y cells. Data represent means ± SD from three independent experiments. **D** Expression of caspase-3 and cleavage caspase-3 assessed at 4 h-MNNG. **E** PAR-immunofluorescence (green) and DAPI (blue) in SH-SY5Y cells at 4h-MNNG. Scale bars: 10 μm. **F** Group quantification of **E** from three independent experiments. **G** AIF-immunofluorescence (red) and DAPI (blue) in SH-SY5Y cells at 4 h-MNNG. Scale bar: 10 μm. **H** Colocalization coefficient between AIF and DAPI measured as Mander’s coefficient. **I** Pearson’s correlation coefficient between AIF and DAPI staining. Significance was determined by one-way ANOVA. **p < 0.01, ***p < 0.001, vs control group; ^#^*p* < 0.05, ^##^*p* < 0.01, ^###^*p* < 0.001, vs MNNG group.

MNNG, a classical DNA-alkylating agent, is widely used for inducing parthanatos [23]. One hour before exposed to MNNG, SH-SY5Y cells were incubated with crocetin (0, 25, 50, 100 μM, Fig. 4A). We discovered that MNNG significantly reduced the cell viability to 68.44 ± 0.08% and increased the level of 8-OHdG to 26.16 ± 3.40 ng/ml, while crocetin or PJ34 pre-incubation improved MNNG-induced SH-SY5Y cells survival and reduced 8-OHdG production (Fig. 4B, C). No cleaved caspase-3 thus the active form of this caspase protease in programmed cell death, was detected in any conditions (Fig. 4D), confirming that the MNNG-induced parthanatos was specific to this type of cell-death pathway. In contrast, 4-h incubation with MNNG increased intracellular PAR expression, and induced AIF nuclear translocation; both were inhibited by crocetin significantly (Fig. 4E–I).

Parthanatos pathway has been characterized with detectable DNA damage and PAR accumulation in the early phase, and massive mitochondria damage and irreversible AIF nuclear translocation in the late phase [10]. We found that 8-OHdG and PAR production increased significantly as early as 15 min after initiating MNNG treatment (Fig. S2A–C), with no increase in nuclear translocation of AIF (Fig. S2D–F). Markedly, crocetin suppressed 8-OHdG generation and PAR formation (Fig. S2A–C) within 15 min of MNNG treatment. Taken together, crocetin showed preventative effects at both the early (15 min) and the later phase of parthanatos (4 h).

### Crocetin reduces the MNNG-induced early phase of oxidative stress by inhibiting NOX2 activation

**Fig. 5:**
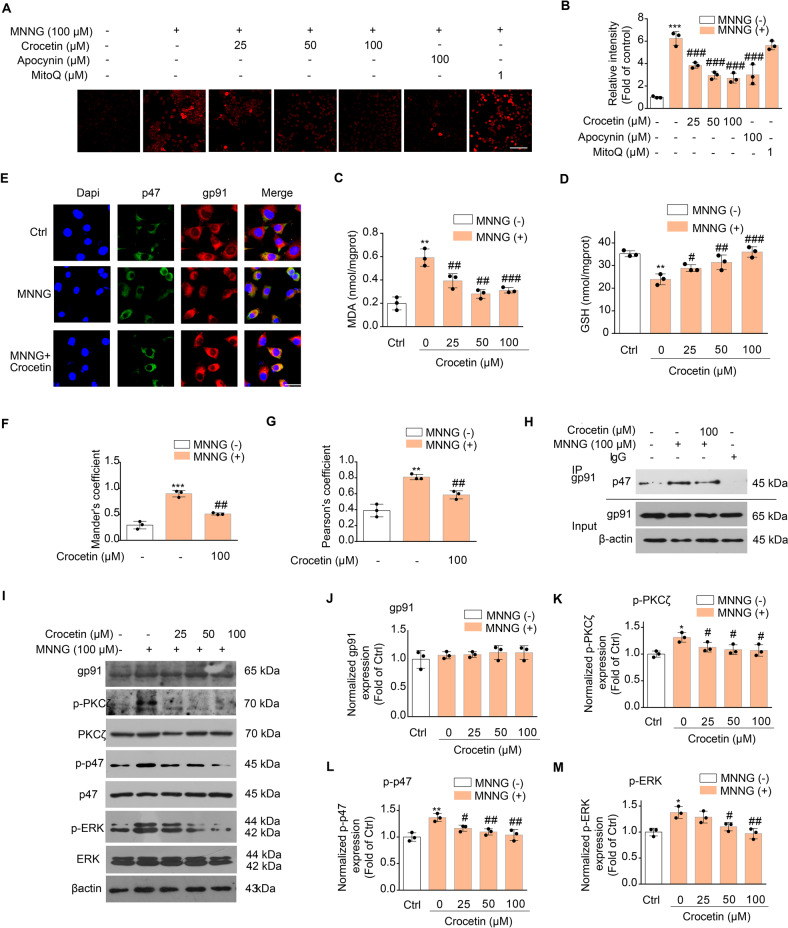
Crocetin reduced ROS production by inhibiting NOX2 activity. **A** Effect of crocetin on MNNG-induced ROS generation (red) in SH-SY5Y cells. Cells were pretreated with crocetin (25, 50, 100 μM) for 1 h and then exposed to MNNG (100 μM) for 15 min. Scale bar: 40 μm. **B** Group quantification of ROS fluorescence intensity in (**A**) from three independent experiments. **C**, **D** Effect of crocetin on MDA level (**C**) and GSH level (**D**) in MNNG-induced early stage of parthanatos. **E** Confocal images of NADPH oxidase subunits p47phox (green) and gp91phox (red) and DAPI (blue). Cells were pretreated with crocetin (100 μM) for 1 h before MNNG (100 μM) stimulation for 15 min. Scale bar: 10 μm. **F** Mander’s coefficient between gp91 and p47 staining. **G** Pearson’s correlation coefficient between gp91 and p47 staining. **H** Co-immunoprecipitation assay shows MNNG-induced binding between p47phox and gp91phox. Cells were pretreated with crocetin (100 μM) for 1 h before MNNG (100 μM) stimulation for 15 min, and cellular lysates were immunoprecipitated using anti-IgG or anti-gp91phox antibodies and immunoblotted using the indicated antibodies. **I** Effect of crocetin on MNNG-induced phosphorylation of PKCζ, p47phox, and ERK. **J**–**M** Group quantification of gp91 (**J**), p-PKCζ (**K**), p-p47 (**L**), p-ERK (**M**) in (**I**) from three independent experiments. Significance was determined by one-way ANOVA. *p < 0.05, **p < 0.01, ***p < 0.001, vs control group; ^#^*p* < 0.05, ^##^*p* < 0.01, ^###^*p* < 0.001, vs MNNG group.

To understand how crocetin inhibits the early phase of parthanatos, we tested antioxidant effect of crocetin. Intracellular superoxide anion (SA) and ROS were measured by DHE and DCFH-DA, respectively [24, 25]. It was observed that MNNG caused a rapid increase in DHE and DCFH-DA fluorescence intensity within 15 min, which was suppressed by pre-treatment of crocetin in a concentration-dependent manner (Fig. 5A, B and Fig. S3A, B). NADPH oxidases inhibitor apocynin significantly reduced the early ROS production while mitochondrial ROS scavengers mitoquinone (MitoQ) has no effect (Fig. 5A, B), supporting that the early ROS was derived from NOX2 but not mitochondria. To further support the ROS measurements, we detected the level of malondialdehyde (MDA), a lipid peroxidation marker [26] and glutathione (GSH), a major intracellular antioxidant [27]. The results showed that crocetin pretreatment not only reduced the MDA levels, but also increased the GSH level (Fig. 5C, D), reflecting the key role of crocetin as an antioxidant in MNNG-induced parthanatos. Correspondingly, Nrf2, a main cellular sensor and regulator of oxidative stress [28, 29], translocated to nuclear in exposure to MNNG due to the increase of ROS, which was similarly inhibited by crocetin treatment (Fig. S3C–E). NADPH oxidases are the key producers of ROS and NOX2/gp91phox is one of the most abundant subunits expressed in neurons [30]. We found that 15-minutes MNNG induced higher colocalization between p47 and NOX2/gp91phox indicating the active oxidase complex to generate ROS, which was suppressed by pre-treatment with crocetin (Fig. 5E–G). We further confirmed the physical interaction between p47 and NOX2/gp91phox, which was suppressed by pre-treatment with crocetin (Fig. 5H). Ferroptosis is related to oxidative stress and lipid peroxidation. However, ferroptosis occurs during the reperfusion but not the ischemic phase [31]. Consistently, glutathione peroxidase 4 (GPX4) and ferritin, two classic markers of ferroptosis, do not change in permanent ischemic stroke rats with or without crocin-treatment (Fig. S4A–C).

Protein kinase Cζ (PKCζ) phosphorylates p47phox and causes membrane-translocation of p47, inducing NOX2 activation [32]. ROS promotes phosphorylation of extracellular signal-regulated kinases (ERK), a subunit of MAPKs [33, 34]. We found that MNNG induced phosphorylation of PKCζ, p47, and ERK, which were all restrained by crocetin pre-treatment in a concentration-dependent manner (Fig. 5I–M). We also found that the expression levels of NOX2/gp91phox, PKCζ, p47, and ERK were unchanged among MNNG- and crocetin-treated conditions, indicating that post-translational mechanisms to activate NOX2 upon MNNG is affected by crocetin pre-incubation (Fig. 5I–M). Collectively, these data demonstrated that crocetin reduced ROS production and the early-phase oxidative stress in neuronal parthanatos by inhibiting PKCζ-mediated NOX2 activation.

### Crocetin inhibits the MNNG-induced late phase of oxidative stress by promoting HK-I binding to mitochondria

**Fig. 6:**
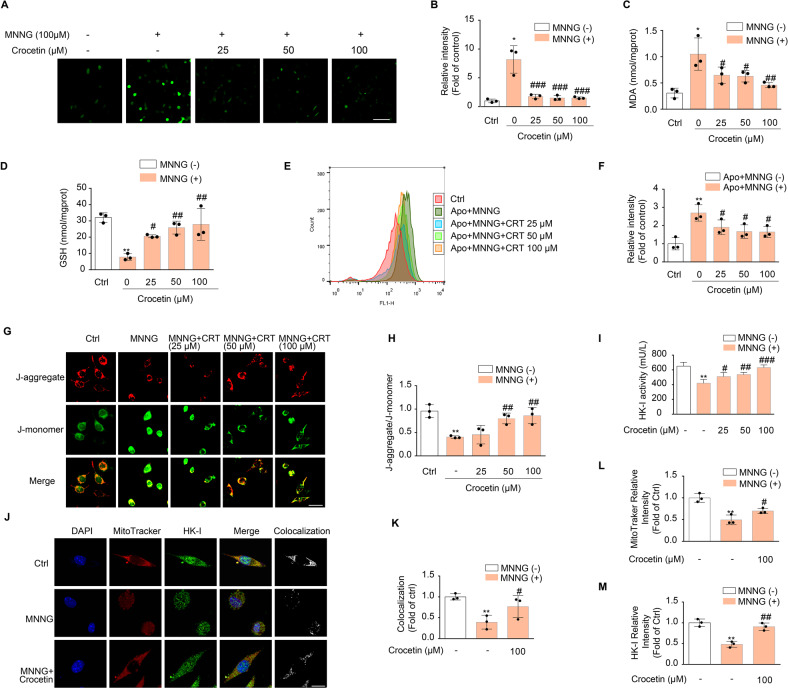
Crocetin preserved mitochondrial HK-I. **A** Effect of crocetin on MNNG-induced ROS generation in SH-SY5Y cells. Cells were pretreated with crocetin (25, 50, 100 μM) for 1 h and then exposed to MNNG (100 μM) for 4 h. Intracellular ROS appeared green under a confocal microscopy (Scale bar is 40 μm). **B** Group quantification of the green fluorescent intensity in (**A**) from three independent experiments. **C**, **D** Effect of crocetin on MDA level (**C**) and GSH level (**D**) in MNNG-induced late stage of parthanatos. **E**, **F** Effect of crocetin on MNNG-induced the late stage of ROS generation. Cells were co-pretreated with indicated concentrations of crocetin and/or apocynin (100 μM) for 1 h, then exposed to MNNG (100 μM) for 4 h. Intracellular ROS was expressed as mean fluorescence intensity which was quantified by flow cytometry. (G) Effect of crocetin on MNNG-induced mitochondria membrane potential in SH-SY5Y cells. Cells were pretreated with indicated concentrations of crocetin for 1 h, then exposed to MNNG (100 μM) for 4 h. JC-1 staining pattern includes J-aggregate(red) at high MMPs and J-monomer(green) at low MMPs. Fluorescence change from red to green indicated a decrease of MMPs. Scale bars: 10 μm. **H** Group quantification of the red and green fluorescent intensity in (**G**) from three independent experiments. **I** Effect of crocetin on MNNG-induced HK-I activity decrease in SH-SY5Y cells. **J** Effect of crocetin on MNNG-induced HK-I release from mitochondria assessed by immunostainings with anti-HK-I (in green) and Mito Tracker Red (in red); nuclei were stained with DAPI (blue). **K** Colocalization between HK-I and mitochondria measured by Image J. **L** Group quantification of the red fluorescent intensity in (**K**) from three independent experiments. **M** Group quantification of the green fluorescent intensity in (**K**) from three independent experiments. Significance was determined by one-way ANOVA. *p < 0.05, **p < 0.01 vs control group; ^#^*p* < 0.05, ^##^*p* < 0.01, ^###^*p* < 0.001, vs MNNG group.

Previous studies have shown that MNNG treatment for 4 h lead to mitochondrial depolarization and the releasing of ROS from mitochondria [6, 11, 32]. To investigate whether crocetin protects mitochondria in the late phase of parthanatos, we measured ROS at the end of a 4-h treatment of MNNG. Significant accumulation of ROS (8.18 ± 2.39 fold of control) upon 4-h MNNG treatment was observed, which was inhibited by the pre-treatment of crocetin in a concentration-dependent manner (Fig. 6A, B). MNNG treatment for 4 h also increased MDA level and decreased GSH level while crocetin pretreatment reversed these effects (Fig. 6C, D). We observed the nuclear translocation of Nrf2 exposed to MNNG for 4 h, which was reversed by crocetin pre-treatment (Fig. S5A–C). To test whether the suppression is due to NOX2 activity being inhibited by crocetin, we pre-treated cells with NADPH oxidases inhibitor apocynin. MNNG-induced ROS accumulation remained in the presence of apocynin, indicating that the ROS overproduction induced by MNNG and its suppression by crocetin in the late phase of parthanatos occurs independently of NOX2 (Fig. 6E, F). We then used MitoTracker to observe mitochondrial morphology and found mitochondrial fragmentation in MNNG-treated cells, which indicates damaged mitochondria associated with cell death. Crocetin significantly improved the intactness of mitochondria, suggesting a protective role of mitochondria by crocetin (Fig. S5D, E). Additionally, MNNG-induced depolarization of mitochondria was prevented by crocetin pre-treatment in a concentration-dependent manner (Fig. 6G, H).

To understand mechanisms underlying the protective effect by crocetin, we focused on glycolysis enzyme hexokinase, whose depletion from mitochondria had been shown to accelerate cell death [19, 35]. We found that HK-I activity was impaired in MNNG-treated cells but restored by crocetin in a concentration-dependent manner (Fig. 6I). Furthermore, fluorescence microscopy showed decreased colocalization between HK-I and MitoTracker upon MNNG stimulation, indicating dissociation of HK-I from mitochondria (Fig. 6J). The dissociation was ameliorated by crocetin, indicated by increased colocalization co-efficient (Fig. 6J, K), as well as HK-I immunostaining intensity at mitochondria (Fig. 6J–M). These data consistently suggested that during the late phase of parthanatos, crocetin alleviates mitochondrial dysfunction and ROS production via promoting HK-I binding to mitochondria.

### Crocetin prevents HK-I degradation mediated by E3 ubiquitin ligase RNF146

**Fig. 7:**
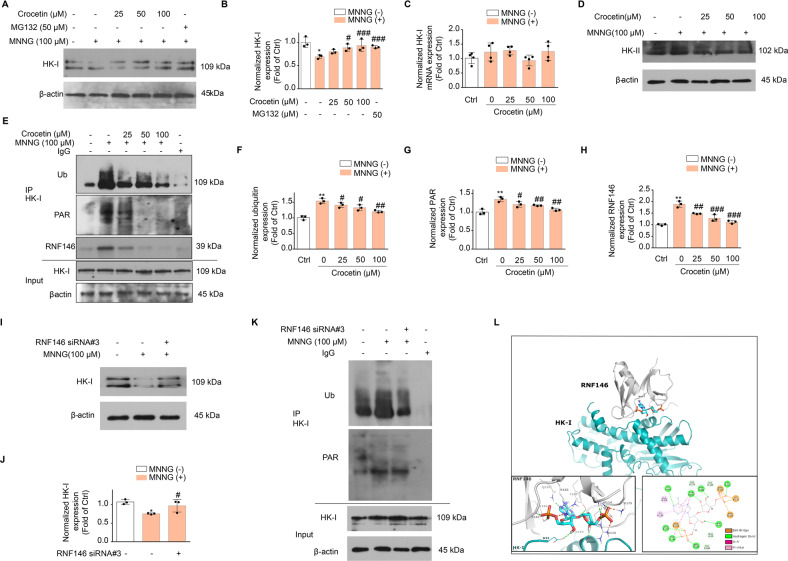
Crocetin reduced E3 ligase RNF146 mediated HK-I ubiquitination and proteasomal degradation. **A** Protein expression of HK-I in SHSY-5Y cells upon MNNG and crocetin-treatment. Cells were pretreated with concentrations of crocetin (25, 50, 100 μM) for 1 h before 4 h-MNNG (100 μM) stimulation. **B** Group quantification of (**A**) from three independent experiments. **C** mRNA expression of HK-I measured using qRT-PCR analysis. Cells were pretreated with concentrations of crocetin (25, 50, 100 μM) for 1 h before MNNG (100 μM) stimulation for 4 h. mRNA expression was normalized to GAPDH mRNA. **D** Protein expression of HK-II in SHSY-5Y cells upon MNNG and crocetin-treatment. **E** PAR, ubiquitination (Ub), and interaction with ubiquitin ligase RNF146 of HK-I protein in SHSY-5Y cells upon MNNG and crocetin-treatment. **F**–**H** Group quantification of Ub (**F**), PAR (**G**), and RNF146 (**H**) in (**E**). Data represent the mean ± SD from three independent experiments. **I** Expression of HK-I protein in SHSY-5Y cells upon RNF146-knock down (siRNA). **J** Group quantification of (**I**). Data represent the mean ± SD from three independent experiments. **K** Effect of RNF146 knock down (siRNA) on the PARylation and ubiquitination level of HK-I. **L** Docking analysis between RNF146 and PARylated HK-I. HK-I was represented in green and RNF146 shown in gray. The interaction types of residue pairs between RNF146 and HK-I were shown in detail. Significance was determined by one-way ANOVA. *p < 0.05, **p < 0.01 vs control group; ^#^*p* < 0.05, ^##^*p* < 0.01, ^###^*p* < 0.001, vs MNNG group.

We found that MNNG reduced HK-I protein expression to 69.09 ± 0.04% without affecting its mRNA level and that crocetin restored HK-I protein expression (Fig. 7A–C), but not HK-II, another predominant isoform of hexokinase in the brain (Fig. 7D). MG132, a proteasome-specific inhibitor, restored HK-I protein expression, suggesting a ubiquitin–proteasomal pathway that degrades HK-I protein during parthanatos.

Post-translational PAR modification serves as a recognition signal for ubiquitin ligases and ubiquitination [36]. Consistently, the ubiquitination and PARylation of HK-I were further found in association with RNF146, an E3 ligase which mediates PARylated substrates degradation [37]. The elevation of ubiquitination in parallel to PAR on the HK-I protein supports the crosstalk between PARylation and ubiquitination pathways. Significantly, pre-treatment of crocetin reduced the ubiquitination and PARylation level on HK-I as well as inhibited the interaction of RNF146 and HK-I (Fig. 7E–H). Knocking down RNF146 led to significant HK-I stabilization after MNNG treatment (Fig. 7I, J and Fig. S6A–C), without affecting PARylation of HK-I (Fig. 7K). Docking simulation between RNF146 and HK-I predicted Lys429 and Arg433 as the key HK-I residues interacting with PAR (Fig. 7L). The molecular docking result corroborated interaction between RNF146 and the PAR-binding motif of HK-I [20]. Furthermore, the participated residues of RNF146 were in the WWE domain, a region responsible for RNF146 to bind to PARylated protein substrates. These data suggested that PARylated HK-I is a novel substrate of RNF146 that targets HK-I for degradation during parthanatos.

### Crocetin directly binds to HK-I and blocks its interaction with RNF146

**Fig. 8:**
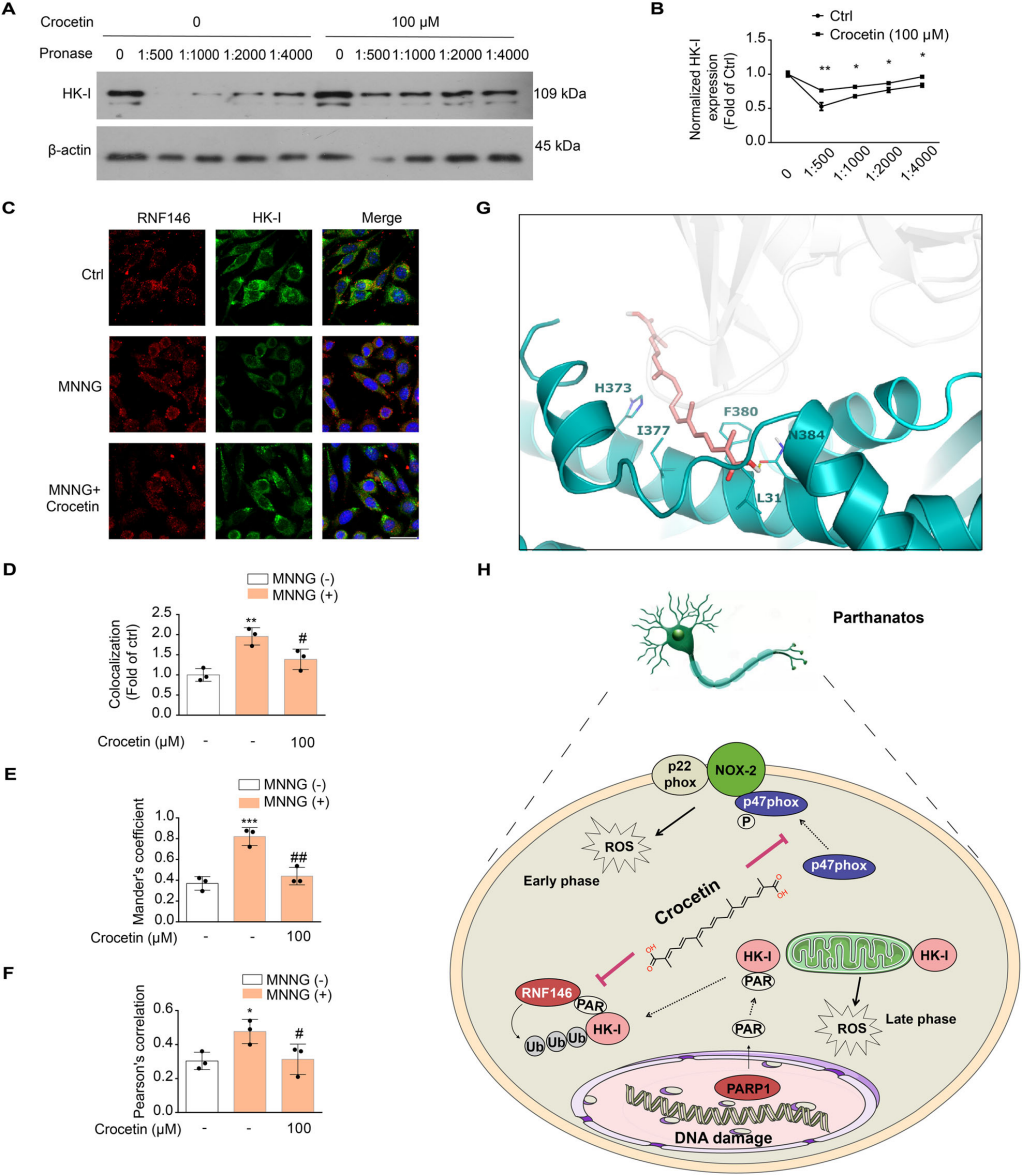
Crocetin bound with HK-I and inhibited HK-I interaction with RNF146. **A** DARTS results from pronase-digested cell lysates. **B** Group quantification of HK-I in (**A**). Data represent the mean ± SD from three independent experiments. **C** Effect of crocetin on MNNG-induced interaction of RNF146 and HK-I by immunofluorescence assay with anti-HK-I (in green) and anti-RNF146 (in red); nuclei were stained with DAPI (blue). Scale bars: 10 μm. **D** Colocalization between HK-I and RNF146 measured by Image J. **E** Mander’s coefficient between HK-I and mitochondria staining. **F** Pearson’s correlation coefficient between HK-I and mitochondria staining. **G** Docking analysis of the crocetin-HK-I complex. **H** The cellular pathways of crocetin to protect neurons from parthanatos and ischemic stroke. Significance was determined by one-way ANOVA. *p < 0.05, **p < 0.01, ***p < 0.001, vs control group; ^#^*p* < 0.05, ^##^*p* < 0.01 vs MNNG group.

To explore how crocetin inhibit HK-I ubiquitination-mediated degradation, we tested possible physical interaction of crocetin and HK-I by carrying out drug affinity responsive target stability (DARTS) assay. We found that the stability of HK-I was enhanced in the crocetin treatment group compared with the control group, suggesting that crocetin might interact with HK-I directly (Fig. 8A, B). Interestingly, the interaction and colocalization between RNF146 and HK-I was significantly weakened by crocetin treatment (Fig. 7E, H and Fig. 8C-F). To elucidate the interaction mode between crocetin and HK-I, molecular docking was performed. As was shown in Fig. 8G and Fig. S7A, the predicted binding residues of HK-I to crocetin were Leu31, His373, Ile377, Phe380, and Asn384. These participated residues were located very closely to the HK-I’s PAR-binding motif that is recognized and bound by RNF146, indicating that crocetin binds to HK-I to impede the recognition of PARylated HK-I by RNF146.

Additionally, HK-I expression was significantly reduced during pMCAO, while oral administration of crocin stabilized HK-I in the brain (Fig. S7B). DARTs assay data from pMCAO rats brain lysates is consistent with that from the cellular lysates, where HK-I protein is protected from protease by crocetin (Fig. S7C).

In conclusion, our study demonstrated that crocetin suppresses MNNG-induced NOX2 activation to reduce ROS generation in early stage of parthanatos and interacts with HK-I to inhibit RNF146-mediated PARylated HK-I degradation in the late stage of parthanatos (Fig. 8H).

## Discussion

Current clinical treatments for acute ischemic stroke are only partially beneficial. Crocetin is a natural apocarotenoid dicarboxylic acid extracted from *C. sativus* and formed by enzymatic deglycosylation of crocin in the intestine [38]. Crocetin possesses multiple pharmacological characteristics, such as antioxidant and anti-inflammatory, with neuroprotective properties in pathological conditions such as ischemic stroke and Alzheimer’s disease [39–43]. Yet, the mechanisms underlying crocetin’s neuroprotective effects are poorly understood; and whether crocetin prevents parthanatos and through what cellular mechanisms are unknown. In the present study, we showed that crocetin prevented parthanatos through two major pathways: (1) inhibiting NOX2 activities to reduce early phase ROS production; (2) inhibiting E3 ligase RNF146-mediated HK-I ubiquitination to protect mitochondria in the later phase of parthanatos.

Early phase of parthanatos is characterized by PAR accumulation after DNA damage and later phase of parthanatos is associated with nuclear translocation of AIF. PAR-binding AIF result in irreversible AIF release from mitochondria into cytosol. AIF in cytosol recruits MIF, a DNA nuclease [44] to enter the nucleus which induces large-scale DNA fragmentation, triggering cell death without caspase-activation [45]. Given the importance of PARP-1 in parthanatos, PARP-1 has been regarded as a potential target to ameliorate ischemic brain injury. However, sex-bias showing protective effects of PARP-1 inhibitors only in male animals has limited their medical applications. Currently no PARP-1 inhibitors are in use for stroke treatment [46]. Based on the crucial role of ROS in DNA damage, antioxidant agents such as propofol have been shown to prevent neuronal parthanatos through reducing ROS-induced intracellular calcium release and attenuating mitochondrial dysfunction. Thus antioxidants may be of particular importance in preventing parthanatos as an IS treatment [11].

Recent studies have shown that NOX2 is the source for early phase ROS production and an upstream signal for PARP-1 in parthanatos [10]. In line with this notion, we found MNNG induced rapid NOX2 activation in early phase. The NOX2-inhibition capacity of crocetin accounted for reducing NOX-derived ROS in early phase. However, it is currently unclear how crocetin inhibits PKCζ-p47phox in detail. One possibility is that crocetin inhibits PKCζ phosphorylation, and another possibility is crocetin suppresses PI3K, which activates PKCζ [47] or decreases Ca^2+^ influx, which acts upstream of PARP-1 activation and DNA damage [48]. Additionally, a previous study showed that crocetin interacted with DNA to form a crocetin–DNA adduct and this adduct may partially protect DNA from oxidative damage [49]. HK is the rate-limiting enzyme for catalyzing glycolysis and interacts with the outer mitochondrial membrane protein VDAC [50]. HK mitochondrial binding protects mitochondrial function and neuron survival from ischemic injury and oxidative damage [19, 51], and further regulates inflammatory responses [52]. It was previously reported that PAR-dependent inhibition of HK-I leads to NAD^+^-independent glycolytic and bioenergetic failure in MNNG-induced parthanatos [21]. In line with this notion, our data showed that MNNG reduced HK-I activity and induced HK-I dissociation from mitochondria. Markedly, crocetin restored HK-I activity and promoted the attachment of HK-I to outer mitochondria membrane, suggesting that the protective effect of crocetin in parthanatos was closely related to preserving HK-I and mitochondrial function.

It was recently reported that altering the subcellular localization of HK-I did not change its enzymatic activity, but shifted glucose flux to pentose phosphate pathway [52]. Our results propose a possibility that this DNA damage-induced glycolytic and bioenergetic failure is due to RNF146-mediated HK-I degradation in parthanatos, which suggests that HK-I is a potential drug target for parthanatos and IS. The RNF146-mediated regulation provided a new mode of HK-I regulation, but how this pathway acts in coordination with other HK-I degradation mechanisms remains to be resolved.

In this study, the dose and administration route of crocin in pMCAO rats were based on the previous studies at non-toxic concentrations [53–55]. The doses we have chosen were much lower than the equivalent doses of crocin used in human studies, where crocin’s effect on brain diseases such as opioid withdrawal syndrome [56] and depressive syndrome [57] was measured, highlighting the potential clinic application value of crocetin on IS. Considerable amount of evidence suggests the roles of parthanatos and HK-I-related bioenergetics failure in neurodegenerative disorders [4, 35, 58]. Our findings suggest that crocetin may have wide-ranging applications in treating ischemic stroke, Alzheimer’s disease, Parkinson’s disease, Amyotrophic Lateral Sclerosis, psychotic disorders and aging.

In summary, we have revealed the anti-parthanaos and anti-oxidative stress effects of crocetin and demonstrated its protecting effect against ischemic stroke in vitro and in vivo, opening a new venue of anti-stroke strategy via inhibiting NOX2 activity and/or preserving mitochondrial HK-I.

## Material and methods

### Reagents

Dulbecco’s minimal essential medium (DMEM), fetal bovine serum (FBS) and 4, 6-diamidino-2-phenylindole (DAPI) were purchased from Sigma Aldrich (St. Louis, MO, USA). Pronase E (HY-11415) was purchased from MedChemExpress (Monmouth Junction, NJ, USA). Crocetin (F0118AS) and RIPA lysis buffer were purchased from meilunbio (Dalian, Liaoning, China). Antibodies for hexokinase-I (DF6199), hexokinase-II (DF6176), NOX2 (DF6520), PKCζ (AF6405), phospho- PKCζ and phosphor-p47phox were purchased from Affinity bioscience (Cincinnati, USA). Antibody for poly (ADP-ribose) polymer (ab14460), GPX4 (ab125066), Ferritin heavy chain (ab183781) and Nrf2 (ab 137550) were purchased from Abcam (Cambridge, MA, USA), Antibodies for lamin B (GB111802), PARP1 (GB111501), and AIF (GB11314) were purchased from Servicebio (Wuhan, Hubei, China). Antibodies for P47 (SC-365215) was purchased from Santa Cruz Biotechnology (Heidelberg, BW, Germany). Antibody for RNF146 (bs-11669) was purchased from Bioss (Beijing, China). Fluorescein (FITC)-conjugated goat anti-mouse IgG H&L (SA00003-1) and Alexa Fluor 594-conjugated goat anti-rabbit IgG H&L (SA00003-4) were obtained from Proteintech (Wuhan, Hubei, China).

### Animals

Six-week-old Sprague–Dawley (SD) rats (male, weighting 180–220 g) were obtained from the Experimental Animal Center of Shenyang Pharmaceutical University (Shenyang, China) and maintained under standard housing conditions in a 12:12 light/dark cycle with access to food and water at libitum. Animal experiments were designed in accordance with the guidelines of the animal facility at Shenyang Pharmaceutical University and approved by Shenyang Pharmaceutical University Animal Care and Use Committee.

### Cell culture

SH-SY5Y cells were obtained from the Cell Bank of the Chinese Academy of Sciences (Shanghai, China). Cells were maintained in DMEM-HAM’S F12, supplemented with 10% fetal calf serum and 1% antibiotics (penicillin, streptomycin) at 37 °C under a humidified 5% CO_2_ atmosphere in an incubator. Cells between passage #5–15 were used.

Cell line has been tested and shown to be no mycoplasma contamination.

### MTT assay

For MTT assay, MTT stock solution (5 mg/mL) was added to the cells to one-tenth of the original culture volume and incubated for 4 h. The purple MTT formazan was solubilized with DMSO and measured by microplate reader (492 nm).

### Oxygen-glucose deprivation

Hypoxia ischemia was simulated by oxygen-glucose deprivation (OGD) as previously described [59]. Briefly, SH-SY5Y cells were seeded at a density of 1 × 10^5^ cell/ml. Twenty-four hour later, cells were pretreated with indicated concentrations of crocetin for 1 h before OGD challenge. Then cells were maintained in the glucose-free culture medium and transferred into a temperature-controlled (37 °C) anaerobic chamber (Billups-Rothenberg Modular Incubator chamber) containing a gas mixture composed of 5% CO_2_ and 95% N_2_ for 2 h. The control group was maintained in normal DMEM in normal incubator for the same incubation time as the OGD cultures.

For oxygen–glucose deprivation and reperfusion (OGD/R) model, the cultures were subjected to reoxytenation by exchanging the medium to glucose restoration and maintained under normoxic conditions for 4 or 24 h, respectively.

### Determination of ROS

SH-SY5Y cells were seeded in 24-well plates at a density of 1 × 10^5^ cells/ml. Twenty-four hour later, cells were treated with various concentrations of crocetin before exposure to MNNG (100 μM) for 15 min or 4 h. Then cells were loaded with 2′, 7′ -dichlorodihydrofluorescein diacetate (DCFH-DA) (5 μM) or dihydroethidium (DHE) (10 μM) in serum-free DMEM for 30 min at 37 °C in the dark. After washing with phosphate-buffered saline (PBS), fluorescence signals were analyzed through confocal microscope. Fluorescence intensity was quantified using Image J.

### Mitochondrial membrane potential (JC-1) assay

Mitochondrial membrane potential changes were measured using JC-1, a fluorescence dye that aggregates in normal mitochondria and disaggregates upon loss of mitochondrial membrane potential. SH-SY5Y cells were seeded in 24-well plates at a density of 1 × 10^5^ cells/well. After treating with various concentrations of crocetin, cells were collected and incubated with JC-1 (10 μM) for 30 min at 37 °C in the dark and rinsed with PBS. Fluorescence images were acquired by confocal microscopy. JC-1 was excited at 488 nm wavelength and emits lights at max wavelengths of 529 nm (monometric form) and 590 nm (J-aggregate form). Fluorescence intensity was quantified using Image J.

### Transfection of small interfering RNA (siRNA)

SH-SY5Y cells were seeded in 6-well plates at a density of 1 × 10^5^ cells/well. 24 h later, transfection of siRNA was performed by using Lipofectamine 8000 (Invitrogen, USA) according to the manufacturer’s instructions. The siRNAs targeting RNF146 were siRNA-1: 5′-GAAGAUUAAGCGAGAUAUATT-3′, siRNA-2: 5′-GGGAGAAGGAGAAGAAGAUTT-3′, and siRNA-3: 5′-CCGUAAACCUAGCAAGAGATT-3′. A negative control (NC) sequence (5′-UUCUCCGAACGUGUCACGUTT-3′) was transfected in parallel cultures. The medium was replaced with fresh DMEM medium 24 h after transfection.

### Western-blot assay

Cells or tissues were homogenized in RIPA lysis buffer supplemented with protease and phosphatase inhibitor cocktails, and the protein concentrations were determined using the Bio-Rad protein assay kit (Hercules, USA). Samples were resolved in SDS-polyacrylamide gels and transferred to polyvinylidene difluoride (PVDF) membranes (EMD Millipore, Billerica, MA, USA). Membranes were blocked with 5% non-fat milk in Tris-buffered saline with 0.1% Tween 20, then incubated with primary antibody overnight at 4 °C. Following washes, membranes were incubated with secondary antibody coupled with horseradish peroxidase for 2 h at room temperature. Immunoblots were developed using enhanced chemiluminescence (ECL) reaction (Thermo Scientific, USA) and imaged on a ChemiDoc XRS System (BioRad, USA). To quantify the changes in protein expression, levels were calculated as follows: (the target protein intensity)/(internal control intensity) using Image J software.

### Immunoprecipitation and co-immunoprecipitation

Immunoprecipitation and co-immunoprecipitation were performed using a Pierce Classic Magnetic IP/Co-IP Kit (Thermo Scientific, USA). Cells were lysed with cold lysis buffer in ice and supernatant was collected after centrifugation at 15,000×*g* for 10 min. Approximately, 500 μg protein was incubated with specific IP antibody (1:25–1:50) at 4 °C on a rotating platform overnight. Pierce Protein A/G Magnetic Beads (25 μL) were added to the antigen sample/antibody mixture and incubated at room temperature for 4 h. After washing, the target antigen–antibody complex was eluted with 100 μL of elution Buffer and 10 μL of Neutralization Buffer, followed by western blotting analysis.

### Immunofluorescence

SH-SY5Y cells (1.0 × 10^5^ cells/well) were seeded on the glass cover slip in 24-well plates and grown for 24 h. Cells were pretreated with crocetin (100 μM) for 1 h before challenging with MNNG (100 μM) [60] for the indicated time points. Cells were washed with PBS and fixed with 4% paraformaldehyde for 20 min at room temperature. Afterwards, cells were permeabilized in PBS containing 0.1% Triton X-100 for 15 min, and blocked with 5% bovine serum albumin for 40 min. Then cells were incubated with specific primary antibodies overnight at 4 °C, followed by incubation with goat Anti-Rabbit IgG H&L (FITC) and/or goat Anti-Mouse IgG H&L Alexa Fluor 594-conjugated for 3 h at room temperature in the dark. After washing, cells were stained with DAPI mounting medium for 7 min. Fluorescence signals were analyzed through confocal microscope.

### Antioxidant measurements

The level of malondialdehyde (MDA) and glutathione (GSH) were analyzed using commercial ELISA (enzyme-linked immunosorbent assay) kits (Jiancheng, Nanjing, Jiangsu, China). In brief, SH-SY5Y cells (1.0 × 10^6^ cells/well) were seeded in 6-well microplates and grown for 24 h. Cells were pretreated with concentrations of crocetin for 1 h before exposure to MNNG (100 mM) for indicated time lengths (15 min or 4 h). At the end of treatments, the cells were collected and processed with ELISA.

### Drug affinity responsive target stability (DARTS)

DARTS assay was performed as described previously [61, 62]. Cells and tissue lysates were obtained from SH-SY5Y cells and SD rat brain, respectively. Cells and tissue were lysed with M-PER lysis buffer (Thermo Scientific, USA). After centrifugation for 10 min at 15,000*g*, the supernatant was obtained, and protein content was quantified using Bio-Rad protein assay kit. Before drug treatment, protein concentration was diluted to 5 μg/μl. Samples were treated with the crocetin (100 μM) or DMSO for 1 h. Samples were then incubated with different dilution ratio pronase (3 mg/ml) or distilled water for 5 min at room temperature. All portion of each sample was used for western blot analysis.

### Docking of RNF146 to HK-I

The protein–protein docking of RNF146 with HK-I was performed using the Haddock 2.2 webserver (http://milou.science.uu.nl/services/HADDOCK2.2). The active amino acid residues were set to HK-I’s residues 420–440, and the passive residues were detected automatically. A score that considers different energies (Van der Waals, electrostatic energies, desolvation and restrains violation energy) is provided at the end of the docking complex analysis. A three-step docking (rigid-body energy minimization, semi-flexible refinement in torsion angle space, and final refinement in explicit solvent refinement) is used to produce a maximum of 100 models of the interaction, which are finally clustered into different groups. Only the cluster which has the best Haddock score is chosen in this work.

### Docking of crocetin to HK-I

HK-I structure (PDB ID 4F9O) was prepared with the protein prepare wizard in Schrodinger package and refined by restrained minimization with the OPLS3e force field. Then the prepared HK-I protein structure was submitted to Discovery Studio 3.0 for molecular-protein docking. During the calculation process, HK-I was set as receptor while crocetin was set as ligand, and the top 10 docking results were reranked according to their docking scores and predicted binding free energy. The top 10 protein-ligand complexes were analyzed with Discovery Studio Visualizer to detect the residues locating at the binding sites which formed non-bond interactions.

### Permanent MCAO in rats

Rats were coded consecutively and segregated randomly into four groups (*n* = 5 per group) based on a random numbers table. Permanent middle cerebral artery occlusion (pMCAO) or sham surgery was performed 5 days after the oral administration of crocin or control. pMCAO was induced using the intraluminal filament model as previously described [63]. Briefly, overnight-fasted rats were anesthetized. A 0.24-mm-diameter poly l-lysine-coated filament with a rounded tip was inserted into the internal carotid artery (ICA) via the right common carotid artery (CCA). Then, the filament was advanced further in the ICA up to the middle cerebral artery until a slight resistance was felt at approximately 18.0 ± 0.5 mm from the carotid artery bifurcation which ensured the occlusion at the origin of the MCA. The filament was fastened around the distal CCA. Finally, the neck incision was closed with sutures. For the sham operation, the rats were subjected to the same procedure except for pMCAO.

### Behavioral tests

The modified neurological severity score (mNSS) test was performed to assess neurological function at 24 h after MCAO as previously described [64]. The test is consisted of motor (muscle status and abnormal movement), sensory (visual, tactile, and proprioceptive), reflexes and balance tests. The mNSS was graded on a scale of 0–18 with higher scores indicating more severity of neurological damage.

### TTC staining

Infarction was evaluated by 2, 3, 5-triphenyltetrazolium chloride (TTC) staining. The brain was harvested, frozen immediately and cut into 2 mm slices throughout the entire brain as previously described [65]. The brain slices were stained with 2% TTC solution (Sigma-Aldrich, St Louis, MO, USA) for 30 min at 37 °C. The infarct volume was measured by Image J software.

### Statistical analysis

Statistical analysis were performed using GraphPad Prism software and SPSS. Values are expressed as mean ± standard deviation (SD). Normality and lognormality test was used. If data did not meet the criteria for normal distribution, non-parametric test was performed. The differences between two groups with similar variances were analyzed using Student’s *t*-test, while one-way ANOVA followed by multiple-comparison tests (LSD) was used to compare the differences among three or more groups. If the variance among groups was not homogeneous, Dunnett’s T3 test was performed. The *p* value lower than 0.05 was considered significant. No statistical method was used to predetermine the sample size for our experiments, which was based on previous experimental observations. Each experiment was repeated at least three times. The sample size of each experiment is shown in the legend. No samples or animals were excluded from the analysis in this work. No blinding method was used. The variance was similar between the groups that were being statistically compared.

### Reporting summary

Further information on research design is available in the Nature Research Reporting Summary linked to this article.

## Supplementary information

an author contribution statement

supplemental figure 1

supplemental figure 2

supplemental figure 3

supplemental figure 4

supplemental figure 5

supplemental figure 6

supplemental figure 7

Supplementary figure legends

Original Data File

Reporting Summary

## Data Availability

All data generated or analyzed during this study are included in this published article and its supplementary information files.
