# Supplementary material for: Crocetin antagonizes parthanatos in ischemic stroke via inhibiting NOX2 and preserving mitochondrial hexokinase-I
Source: Cell Death Dis. 2023 Jan 21;14(1):50. doi: 10.1038/s41419-023-05581-x (PMC9867762; doi:10.1038/s41419-023-05581-x)
Supplement: Supplementary file 1 — an author contribution statement [file 41419_2023_5581_MOESM1_ESM.docx]

**Author contributions**

Hao Wu, Wanxu Huang and Dan Ohtan Wang conceived the work. Hao Wu participated in the design and implementation of the experiment. Hao Wu, Ying Li, Qian Zhang, Hanxun Wang, Wenyu Xiu, Yujie Deng, Pu Xu carried out data processing and analysis. Hao Wu, Wanxu Huang and Dan Ohtan Wang wrote and modified paper. All authors discussed and approved the manuscript.
