# Supplementary figures and images for: Crocetin antagonizes parthanatos in ischemic stroke via inhibiting NOX2 and preserving mitochondrial hexokinase-I
Source: Cell Death Dis. 2023 Jan 21;14(1):50. doi: 10.1038/s41419-023-05581-x (PMC9867762; doi:10.1038/s41419-023-05581-x)

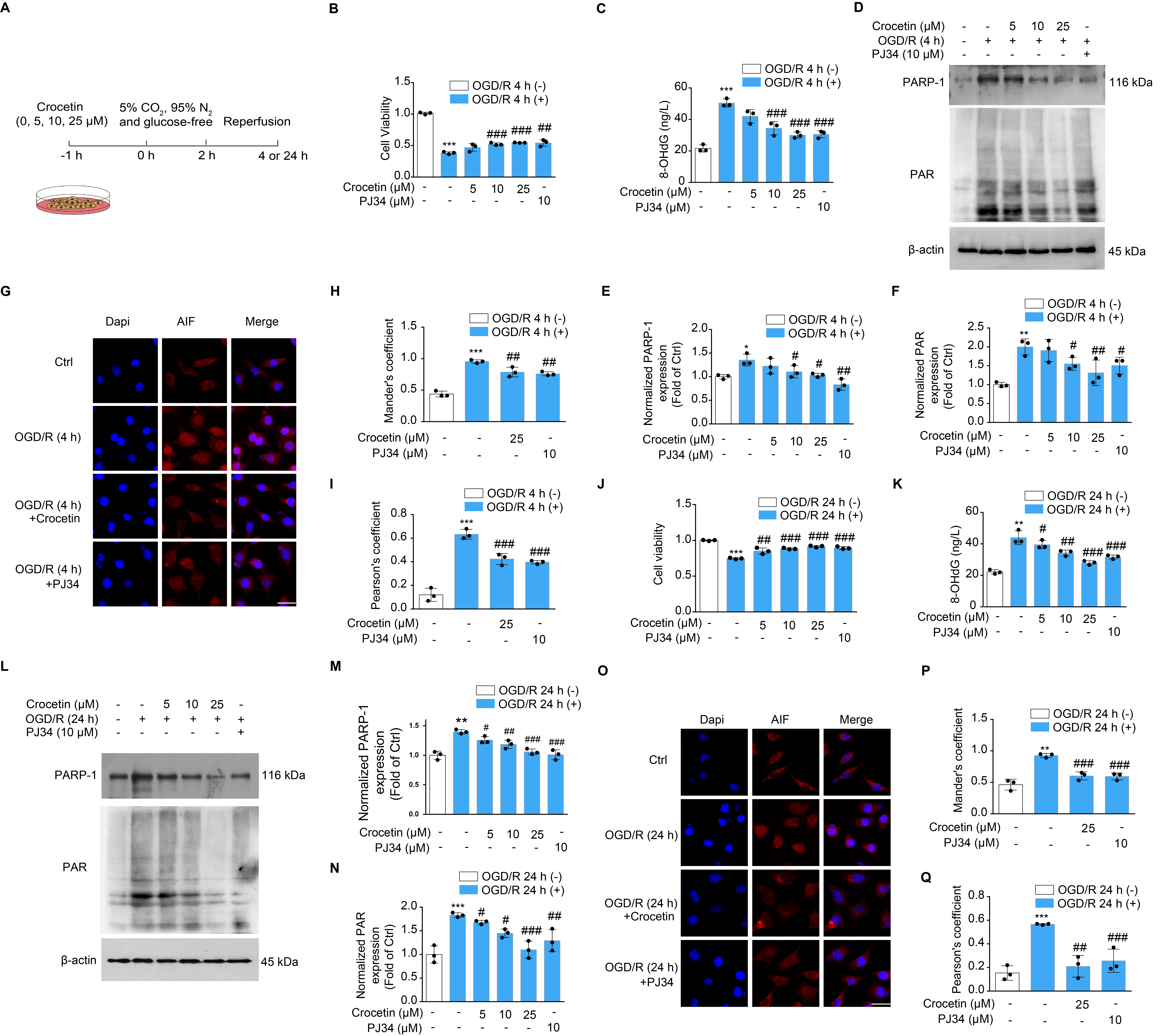

Supplement: Supplementary file 2 — supplemental figure 1 [file 41419_2023_5581_MOESM2_ESM.jpg]

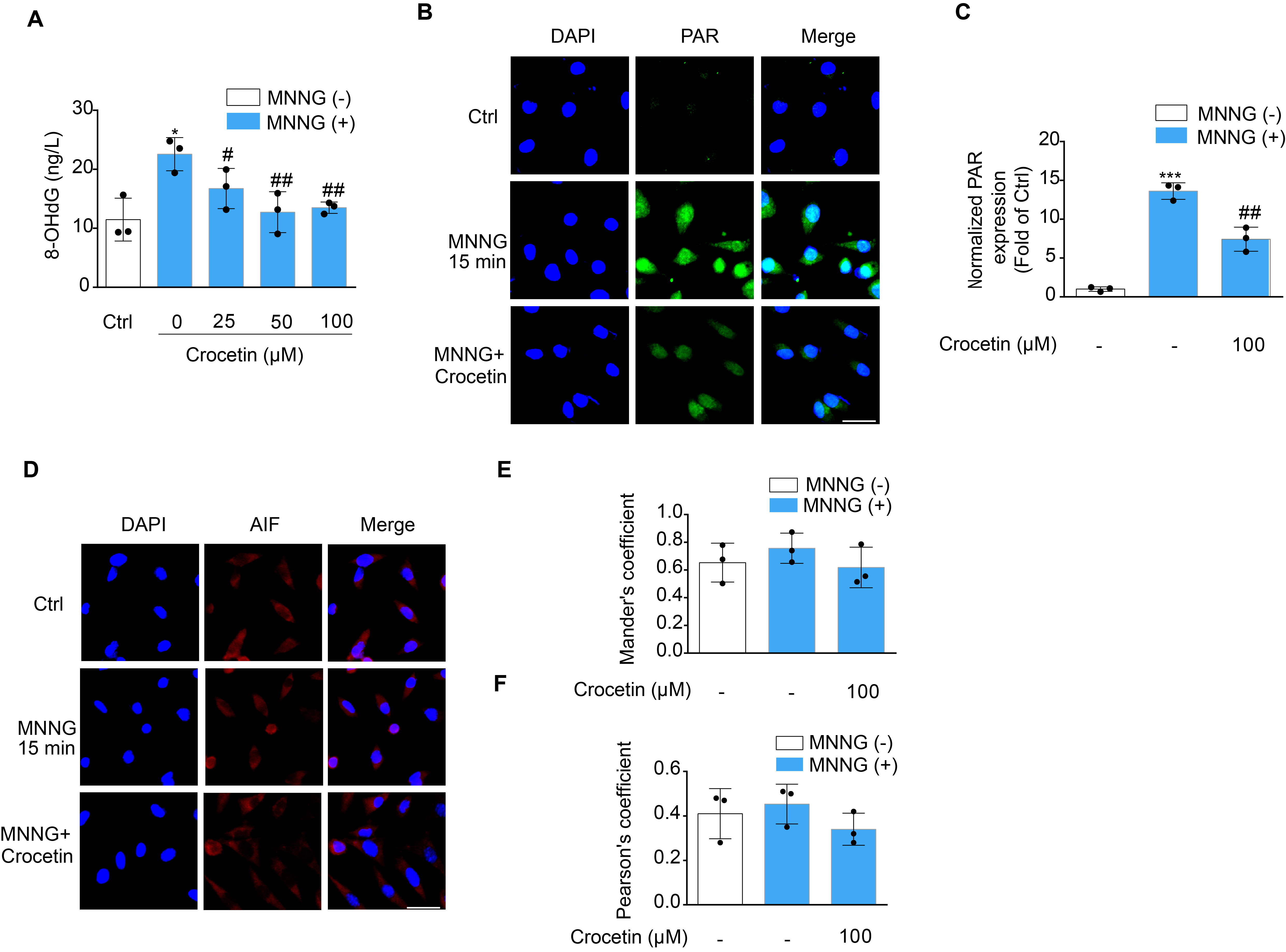

Supplement: Supplementary file 3 — supplemental figure 2 [file 41419_2023_5581_MOESM3_ESM.jpg]

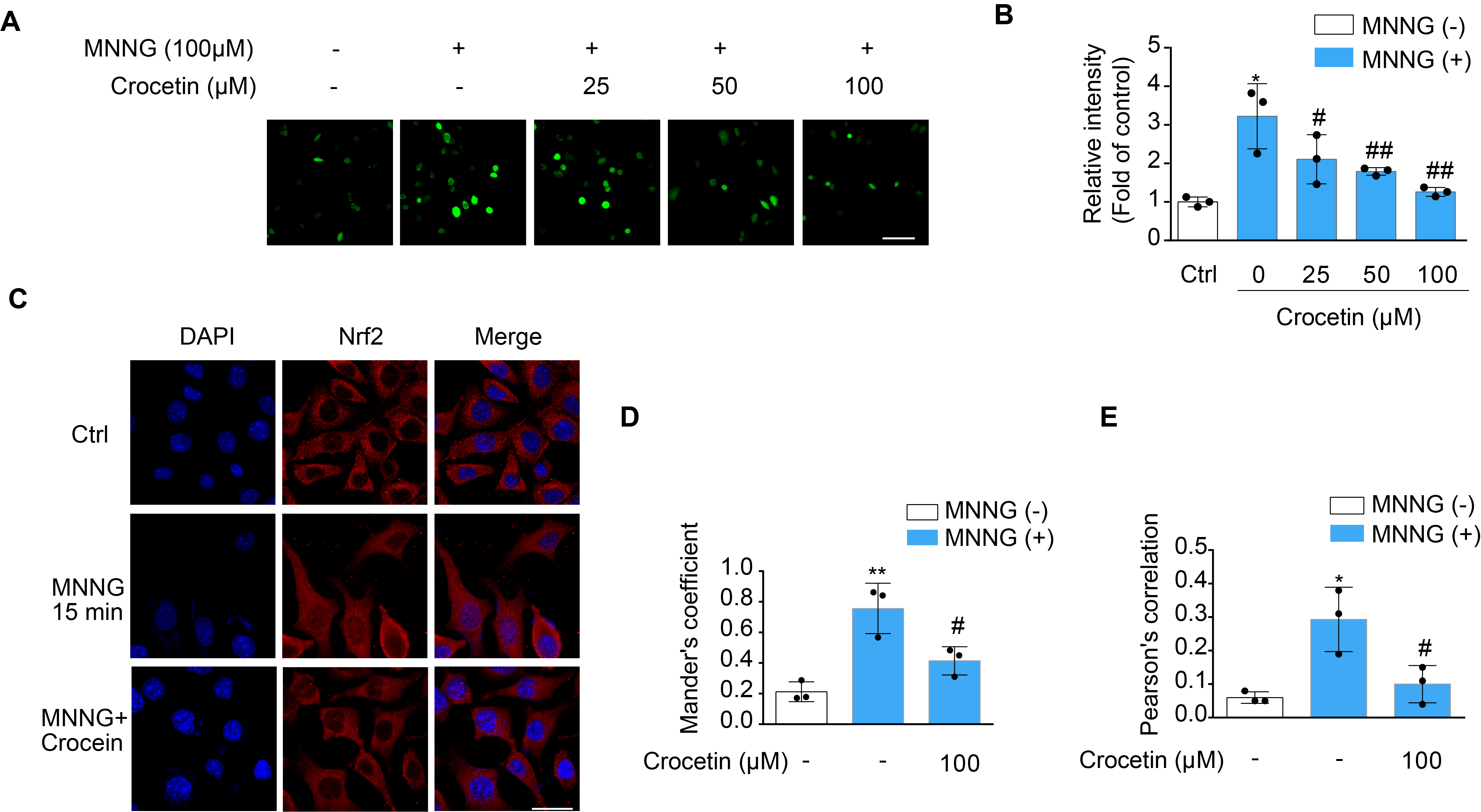

Supplement: Supplementary file 4 — supplemental figure 3 [file 41419_2023_5581_MOESM4_ESM.jpg]

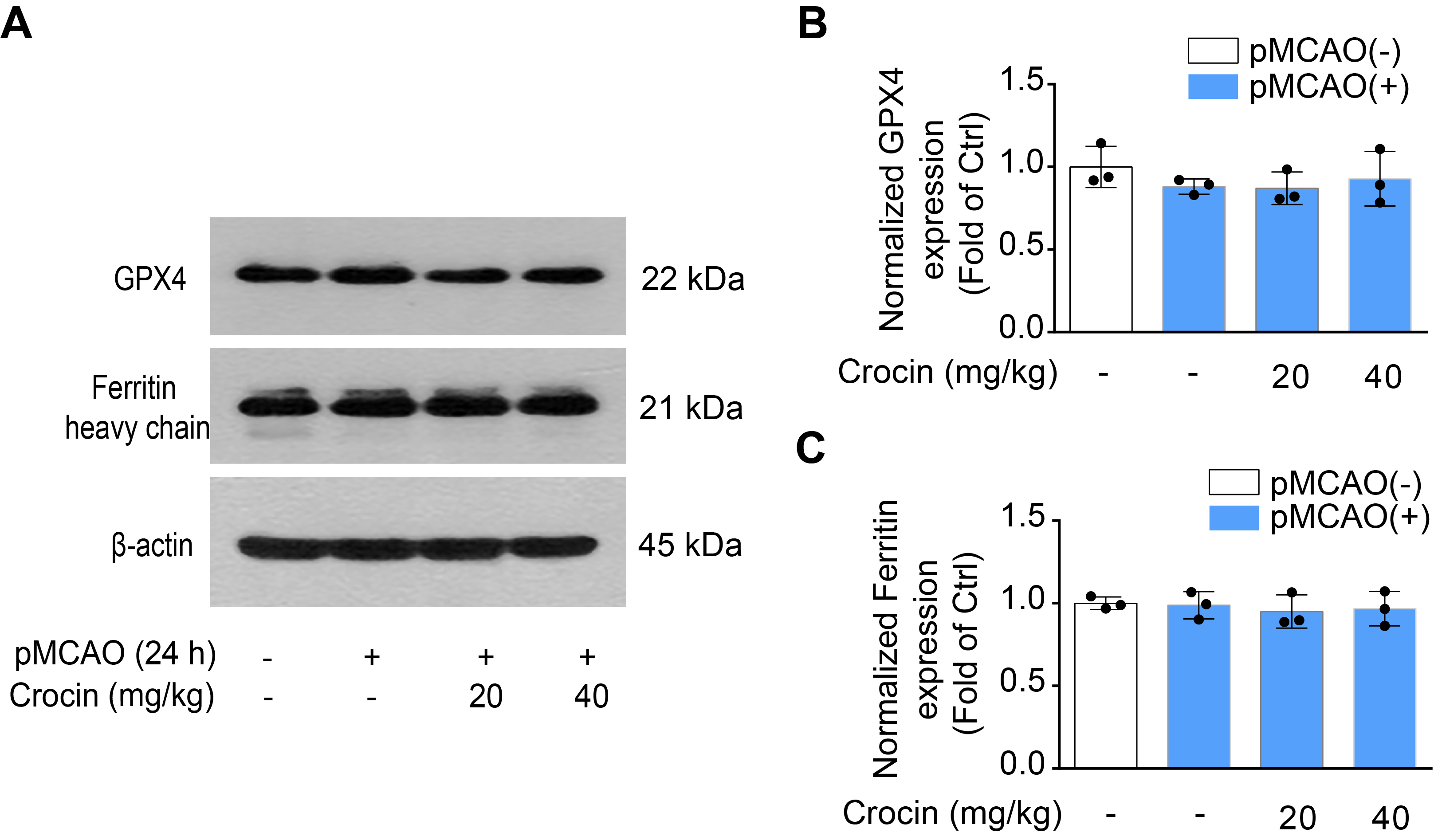

Supplement: Supplementary file 5 — supplemental figure 4 [file 41419_2023_5581_MOESM5_ESM.jpg]

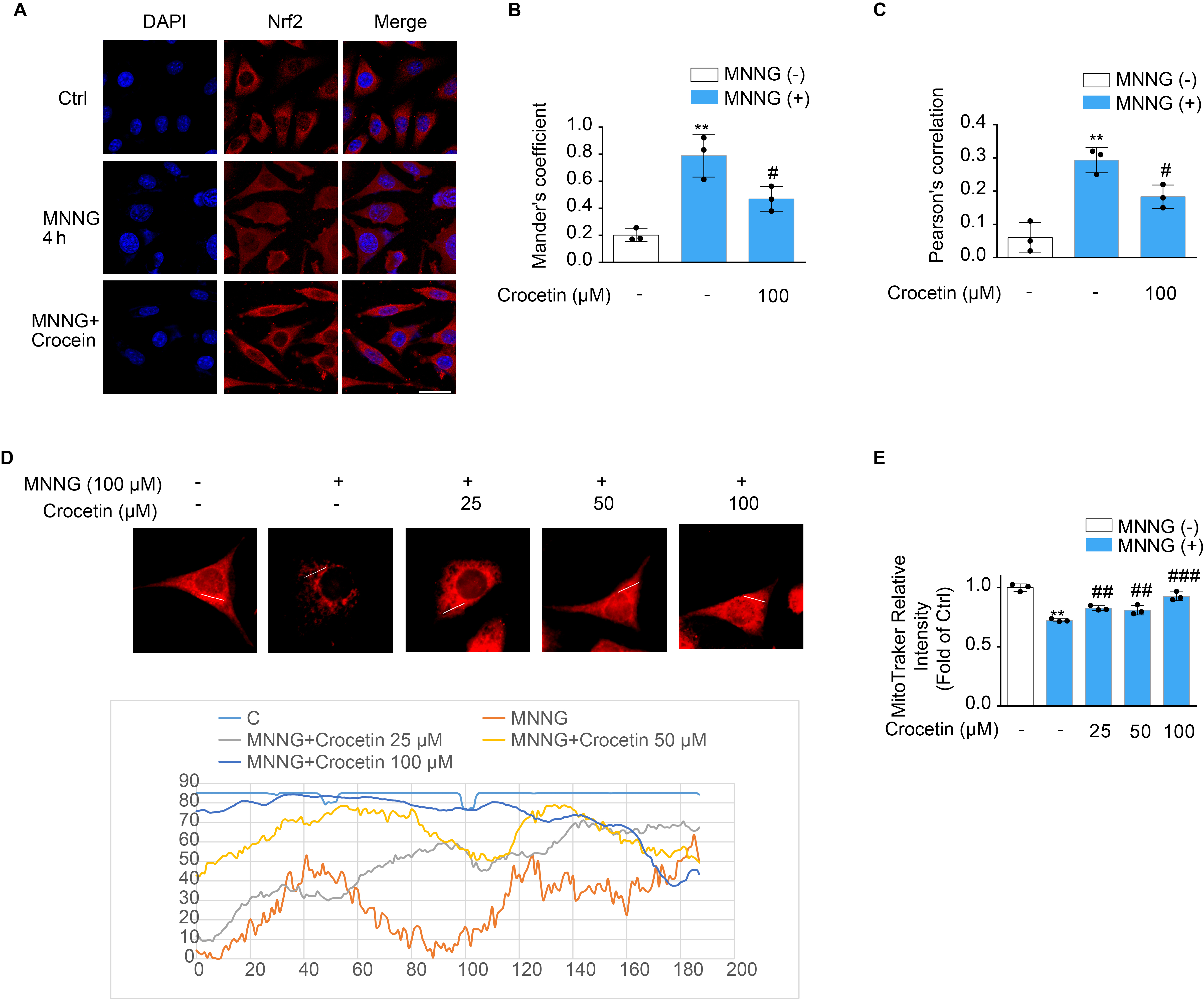

Supplement: Supplementary file 6 — supplemental figure 5 [file 41419_2023_5581_MOESM6_ESM.jpg]

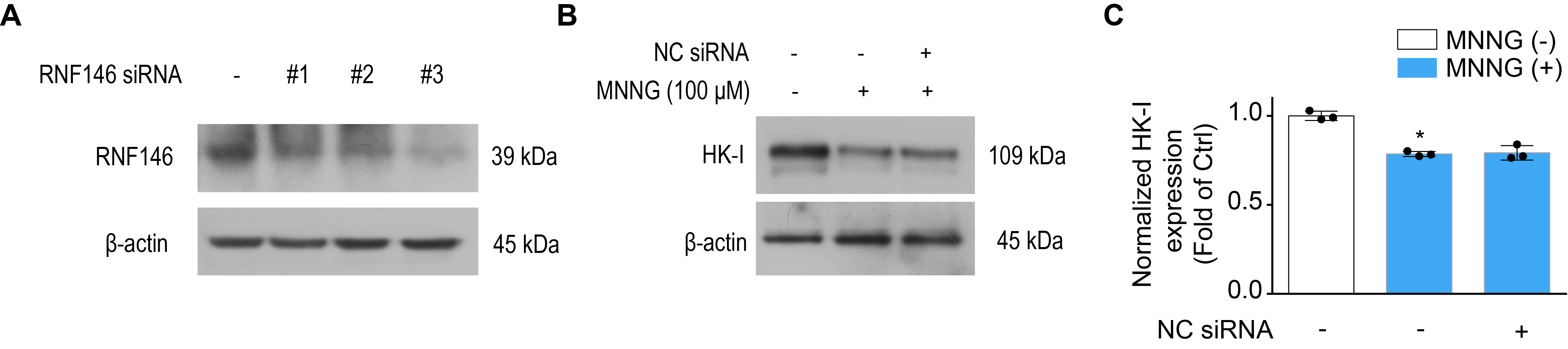

Supplement: Supplementary file 7 — supplemental figure 6 [file 41419_2023_5581_MOESM7_ESM.jpg]

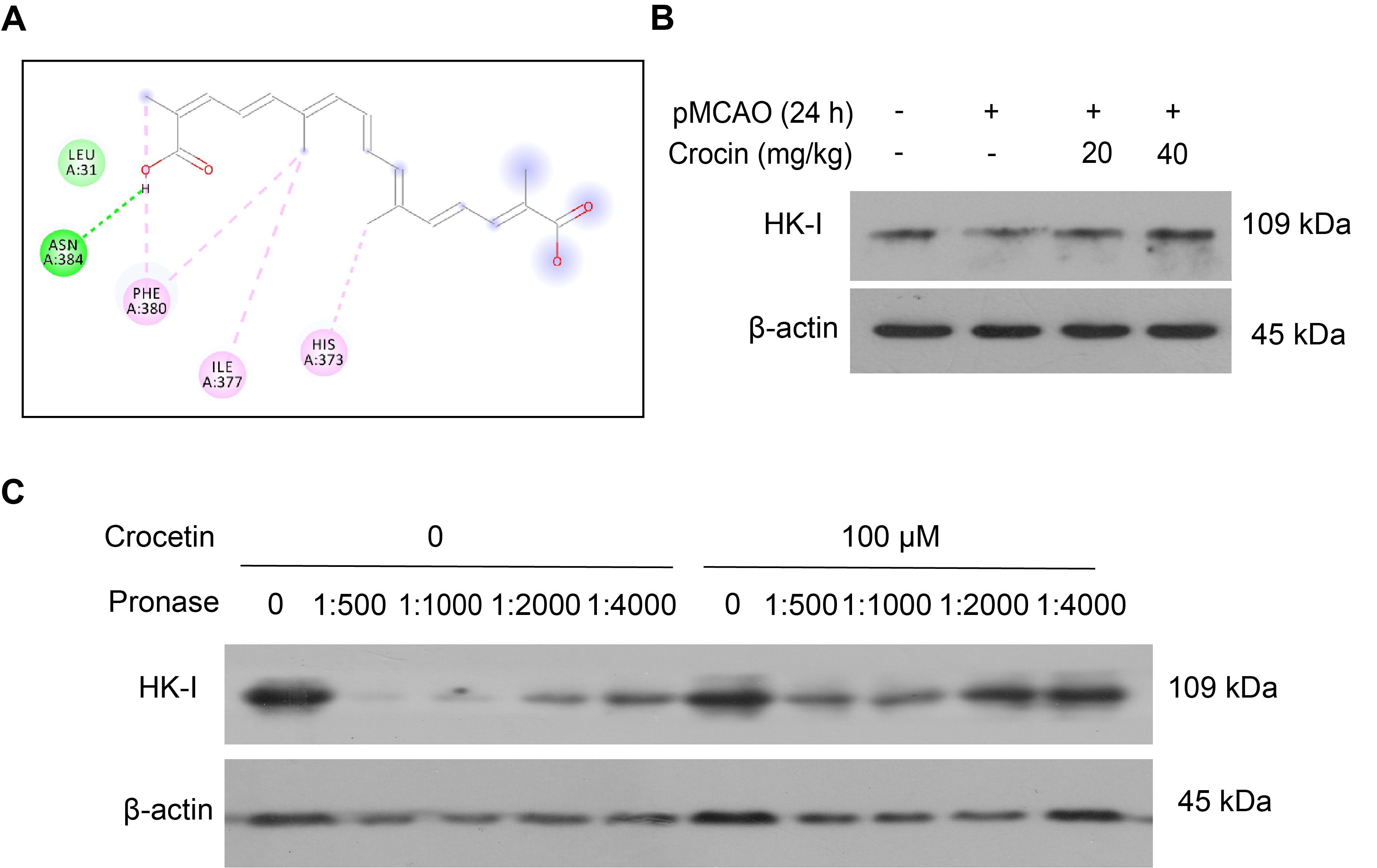

Supplement: Supplementary file 8 — supplemental figure 7 [file 41419_2023_5581_MOESM8_ESM.jpg]
