# Supplementary material for: Crocetin antagonizes parthanatos in ischemic stroke via inhibiting NOX2 and preserving mitochondrial hexokinase-I
Source: Cell Death Dis. 2023 Jan 21;14(1):50. doi: 10.1038/s41419-023-05581-x (PMC9867762; doi:10.1038/s41419-023-05581-x)
Supplement: Supplementary file 9 — Supplementary figure legends [file 41419_2023_5581_MOESM9_ESM.docx]

**Figure S1. Anti-pathanatos effects of crocetin on the secondary cell damage.**

(A) Scheme diagram of the experiment. SH-SY5Y cells were treated with indicated concentrations of crocetin or PJ34 (10 μM) for 1 h before 2 h-OGD. After OGD, the cells were subjected to reoxytenation by exchanging the medium to glucose restoration and maintained under normoxic conditions for 4 h or 24 h respectively (there is no crocetin or PJ34 treatment during OGD/R). (B) Cell viability was determined with MTT assay. (C) OGD/R (4 h)-induced 8-OHdG in SH-SY5Y cells. (D) OGD/R (4 h)-induced PARP-1 and PAR expression SH-SY5Y cells upon crocetin or PJ34 pre-treatment. (E) Group quantification of PARP-1 in (D) from three independent experiments. (F) Group quantification of PAR in (D) from three independent experiments. (G) Confocal images of SH-SY5Y cells immuno-stained with AIF-specific antibody (red) and DAPI (blue). Scale bar: 10 μm. (H) Colocalization coefficient between AIF and DAPI measured as Mander’s coefficient. (I) Pearson’s correlation coefficient between AIF and DAPI staining. (J) Cell viability was determined with MTT assay. (K) OGD/R (24 h)-induced 8-OHdG in SH-SY5Y cells. (L) OGD/R (24 h)-induced PARP-1 and PAR expression SH-SY5Y cells upon crocetin or PJ34 pre-treatment. (M) Group quantification of PARP-1 in (L) from three independent experiments. (N) Group quantification of PAR in (L) from three independent experiments. (O) Confocal images of SH-SY5Y cells immuno-stained with AIF-specific antibody (red) and DAPI (blue). Scale bar: 10 μm. (P) Colocalization coefficient between AIF and DAPI measured as Mander’s coefficient. (Q) Pearson’s correlation coefficient between AIF and DAPI staining. Data represent the mean ± SD from three independent experiments. Significance was determined by one-way ANOVA. *p < 0.05, **p < 0.01, ***p < 0.001, vs. control group; ^#^p < 0.05, ^##^p < 0.01, ^###^p < 0.001, vs. OGD group.

**Figure S2. Anti-pathanatos effects of crocetin at early pathanatotic phases.**

(A) 8-OHdG expression (ng/L) in SH-SY5Y cells at 15 min-MNNG. Data represent means ± SD from three independent experiments. (B) PAR-immunofluorescence (green) and DAPI (blue) in SH-SY5Y cells at 15 min-MNNG. Scale bars: 10 μm. (C) Group quantification of (B) from three independent experiments. (D) AIF-immunofluorescence (red) and DAPI (blue) in SH-SY5Y cells at 15 min-MNNG. Scale bar: 10 μm. (E) Mander’s coefficient between AIF and DAPI staining. (F) Pearson’s correlation coefficient between AIF and DAPI staining. Significance was determined by one-way ANOVA. *p < 0.05, **p < 0.01, ***p < 0.001, vs. control group; ^#^p < 0.05, ^##^p < 0.01, ^###^p < 0.001, vs. MNNG group.

**Figure S3. Crocetin inhibited ROS generation and Nrf2 translocation at early pathanatotic phases.**

(A) Effect of crocetin on MNNG-induced ROS generation (green) in SH-SY5Y cells. Cells were pretreated with crocetin (25, 50, 100 μM) for 1 h and then exposed to MNNG (100 μM) for 15 min. Scale bar: 40 μm. (B) Group quantification of red fluorescence intensity in (A) from three independent experiments. (C) Nrf2-immunofluorescence (red) and DAPI (blue) in SH-SY5Y cells at 15 min-MNNG. Scale bar: 10 μm. (D) Mander’s coefficient between Nrf2 and DAPI staining. (E) Pearson’s correlation coefficient between Nrf2 and DAPI staining. Significance was determined by one-way ANOVA. *p < 0.05, **p < 0.01, ***p < 0.001, vs. control group; ^#^p < 0.05, ^##^p < 0.01, ^###^p < 0.001, vs. MNNG group.

**Figure S4. Effect of crocin on GPX4 and Ferritin levels**

(A) Western blot of GPX4 and Ferritin. (B) Quantification of GPX4 in (A) from three independent experiments. (C) Quantification of Ferritin in (A) from three independent experiments. Data represent the mean ± SD from three independent experiments.

**Figure S5. Crocetin ameliorated mitochondria injury and reversed Nrf2 translocation**

(A) Nrf2-immunofluorescence (red) and DAPI (blue) in SH-SY5Y cells at 4 h-MNNG. Scale bar: 10 μm. (B) Mander’s coefficient between Nrf2 and DAPI staining. (C) Pearson’s correlation coefficient between Nrf2 and DAPI staining. (D) Effect of crocetin on MNNG-induced mitochondria damage in SH-SY5Y cells. MitoTracker Red represented the mitochondria injury under a confocal microscopy (Scale bar is 5 μm). Mean red fluorescence intensity was analyzed by Image J. (E) Group quantification of the red fluorescent intensity in (D) from three independent experiments. Data represent the mean ± SD from three independent experiments. Significance was determined by one-way ANOVA. *p < 0.05, **p < 0.01, ***p < 0.001, vs. control group; ^#^p < 0.05, ^##^p < 0.01, ^###^p < 0.001, vs. MNNG group.

**Figure S6. Knock down efficiency of three RNF146 siRNA and effect of NC siRNA on MNNG induced HK-I expression reduction.**

(A) HK-I expression in SHSY-5Y cells upon three different siRNA transduction (72 h). (B) HK-I expression in SHSY-5Y cells upon NC siRNA transduction (72 h). (C) Group quantification of (B). Data represent the mean ± SD from three independent experiments. Significance was determined by one-way ANOVA. *p < 0.05, **p < 0.01, ***p < 0.001, vs. control group; ^#^p < 0.05, ^##^p < 0.01, ^###^p < 0.001, vs. MNNG group.

**Figure S7. Crocin stabilized HK-I expression in pMCAO rats through metabolizing to crocetin then binding with HK-I**

(A) Docking analysis of the crocetin-HK-I complex. (B) Western blot was performed with anti-HK-I. Loading control, β-actin. (C) DARTS results from pronase-digested brain lysates. The rat brain lysates were prepared with crocetin (100 μM) or DMSO for 1 h, followed by digestion with pronase.
