# Supplementary material for: Crocetin antagonizes parthanatos in ischemic stroke via inhibiting NOX2 and preserving mitochondrial hexokinase-I
Source: Cell Death Dis. 2023 Jan 21;14(1):50. doi: 10.1038/s41419-023-05581-x (PMC9867762; doi:10.1038/s41419-023-05581-x)

Fig.1

Ctrl. MCAO, MCAO+crocin(20, 40 mg/kg).

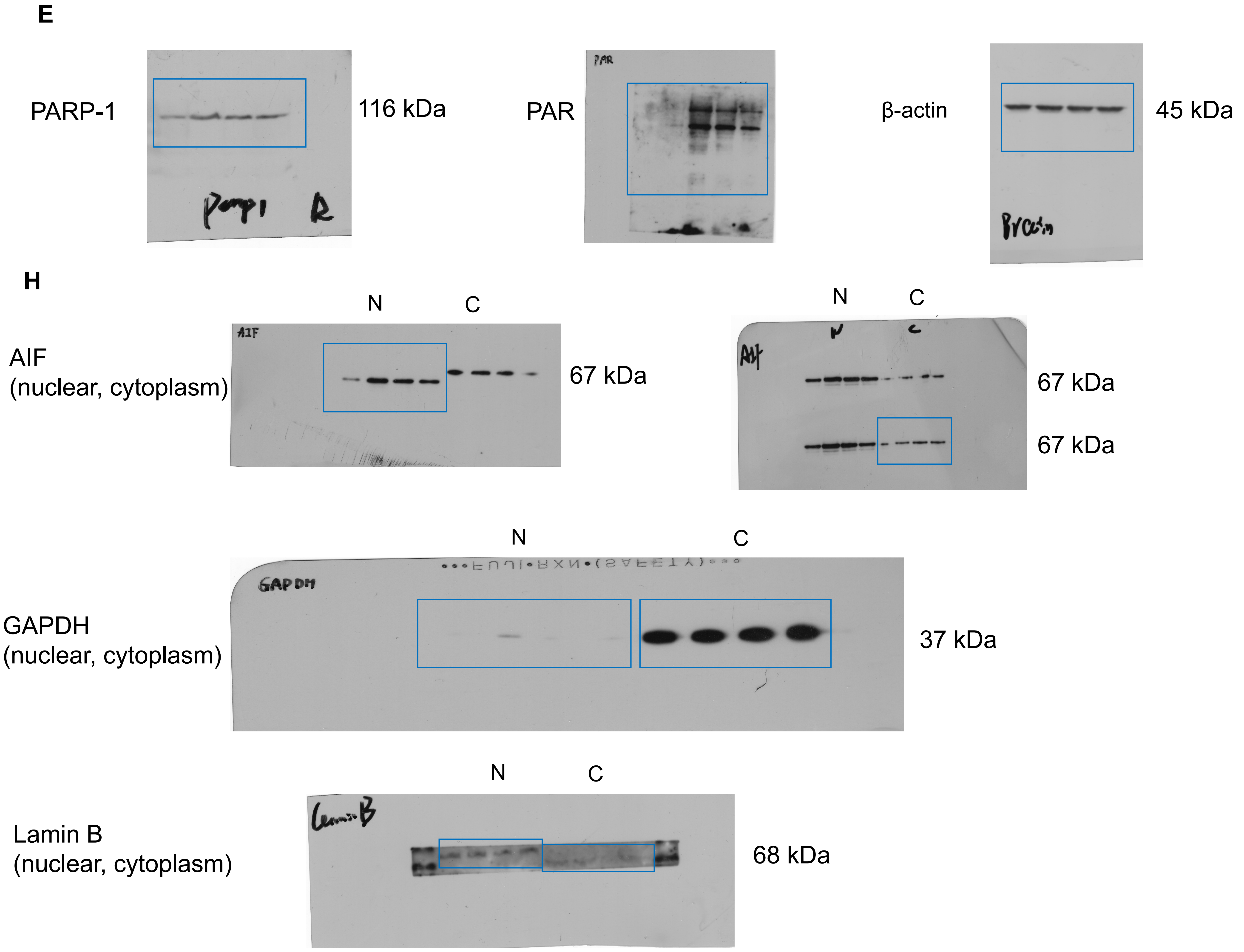

Fig.2

Ctrl, OGD. OGD+crocetin (5, 10, 25 μM), OGD+PJ34

D

PARP-1

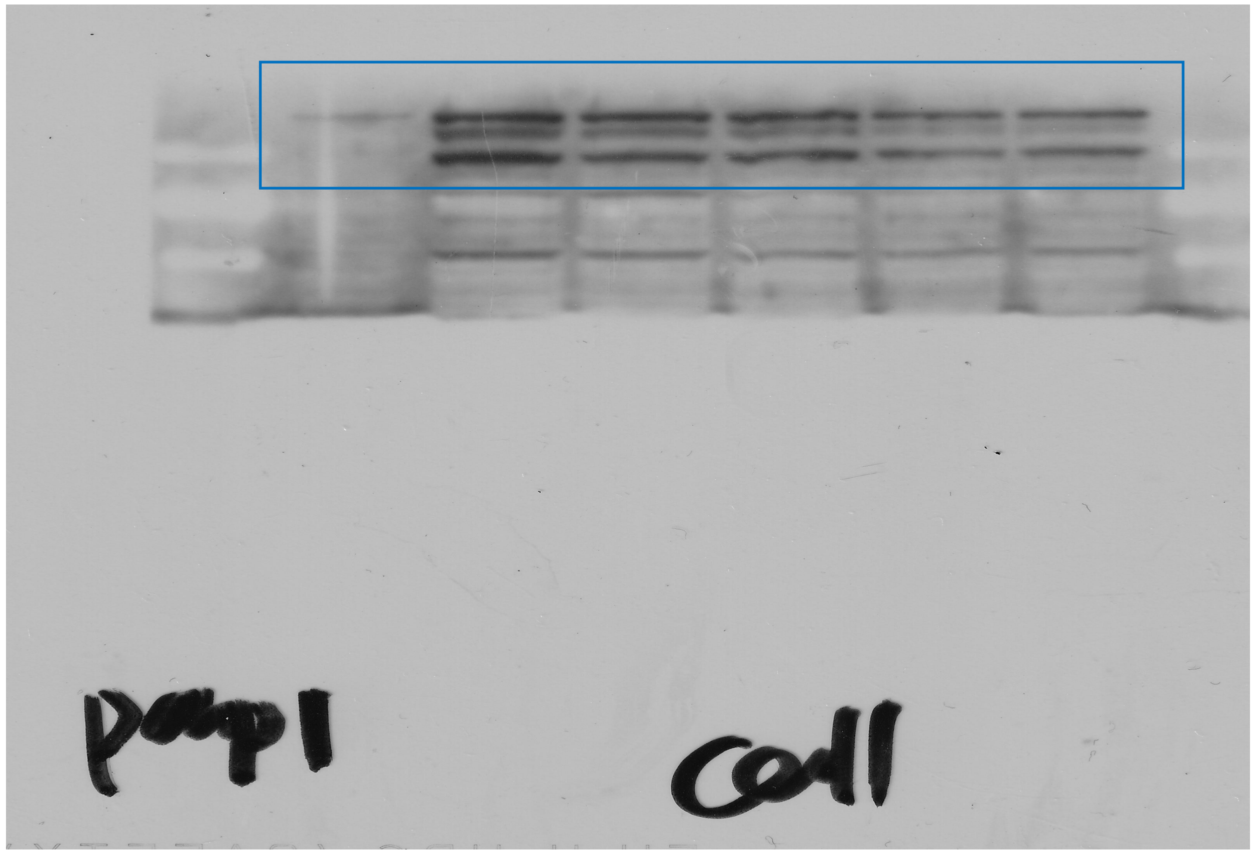

116 kDa

β-acint

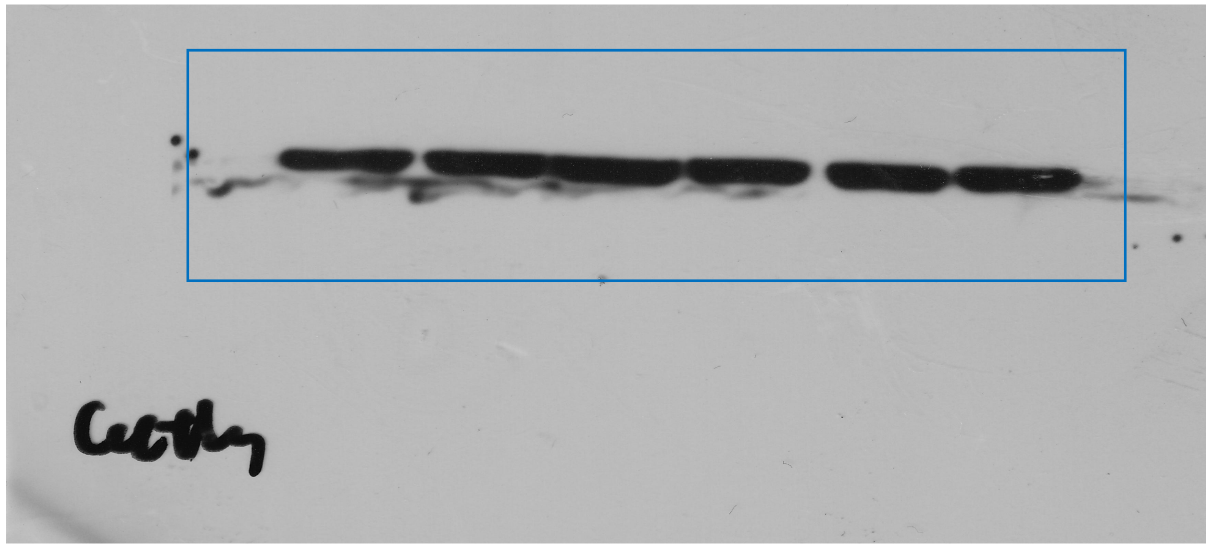

45 kDa

Fig.3

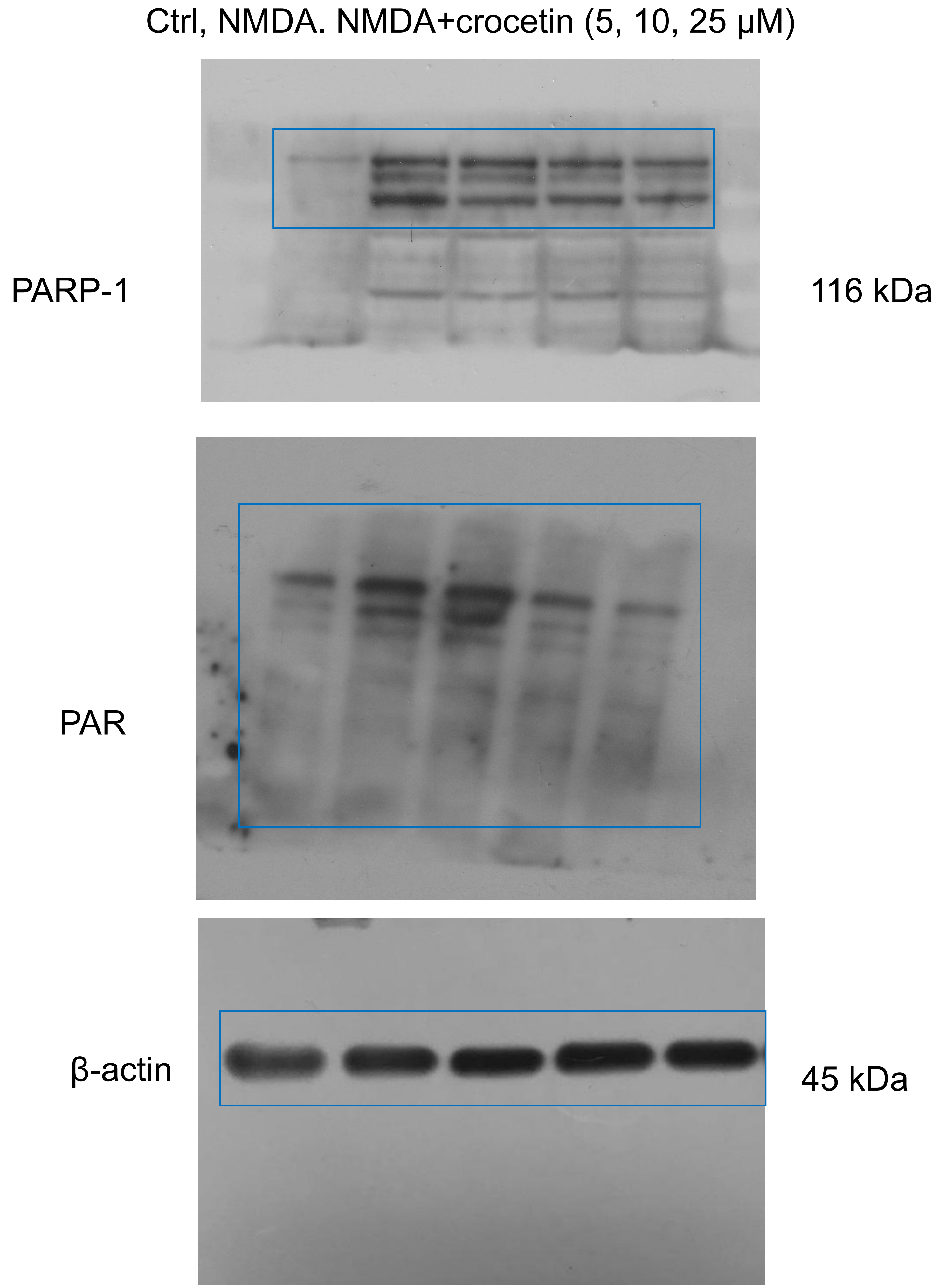
Ctrl, NMDA. NMDA+crocetin (5, 10, 25 μM)
D
PARP-1
116 kDa
PAR
β-actin
45 kDa

Fig. 4

Ctrl, MNNG. MNNG+crocetin (25, 50, 100 μM)

D

Caspase-3 — 35 kDa

cleaved-Caspase-3 — 17 kDa, 15 kDa

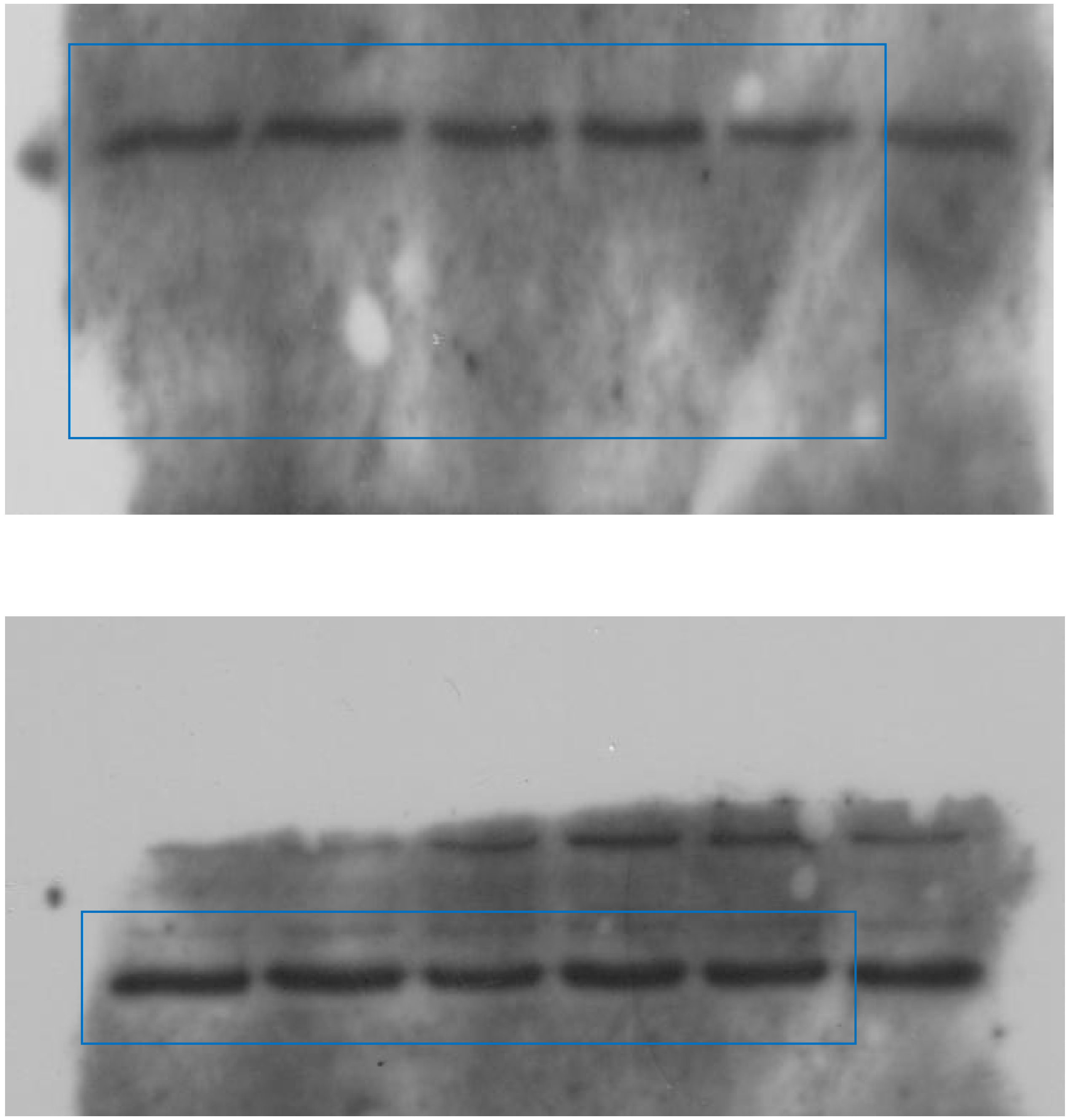

β-actin — 45 kDa

Fig. 5

H

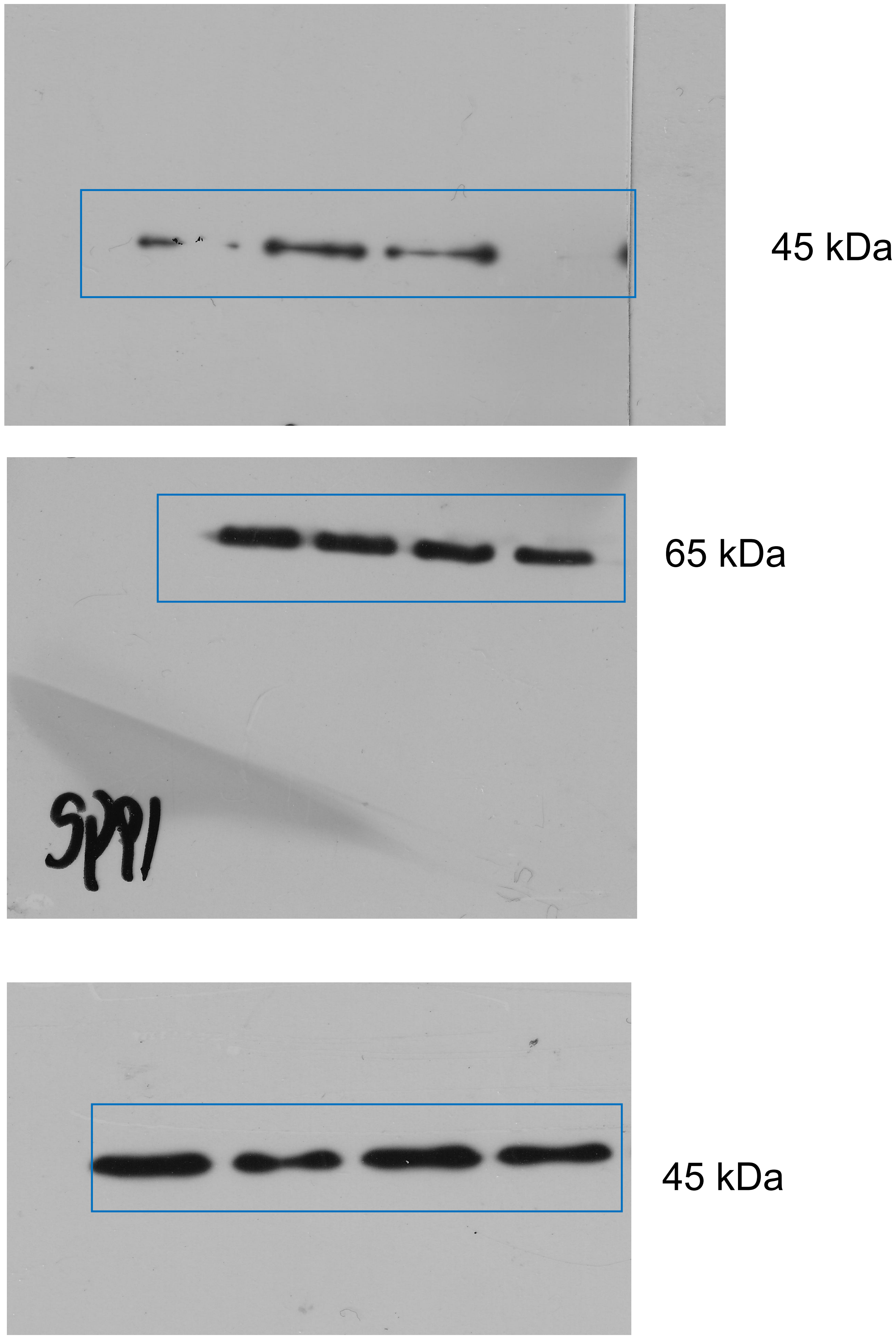
Crtl, MNNG. MNNG+crocetin, IgG
p47
45 kDa
gp91
65 kDa
β-actin
45 kDa

I

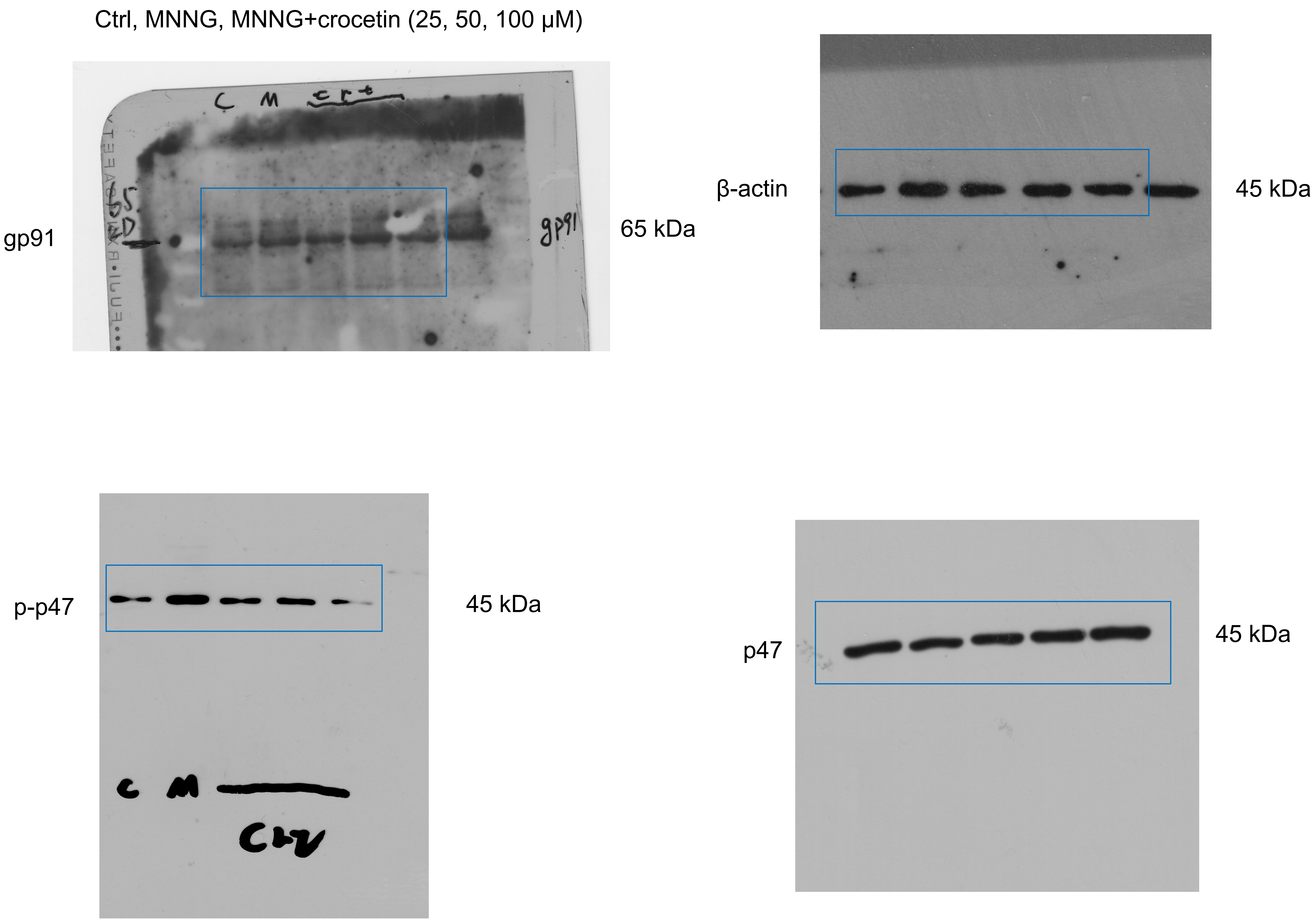
Ctrl, MNNG, MNNG+crocetin (25, 50, 100 μM)
gp91
65 kDa
β-actin
45 kDa
p-p47
45 kDa
p47
45 kDa

Ctrl, MNNG, MNNG+crocetin (25, 50, 100 μM)

p-ERK

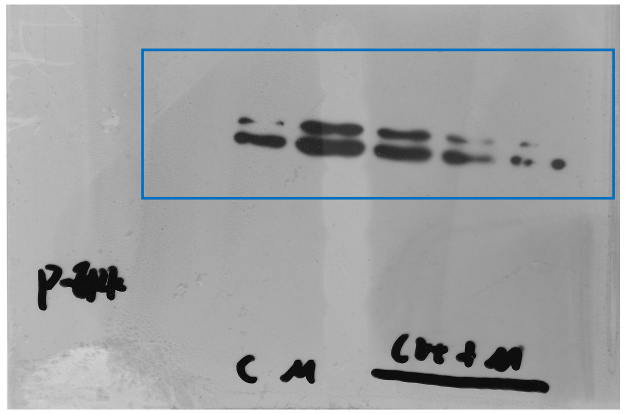

44 kDa
42 kDa

ERK

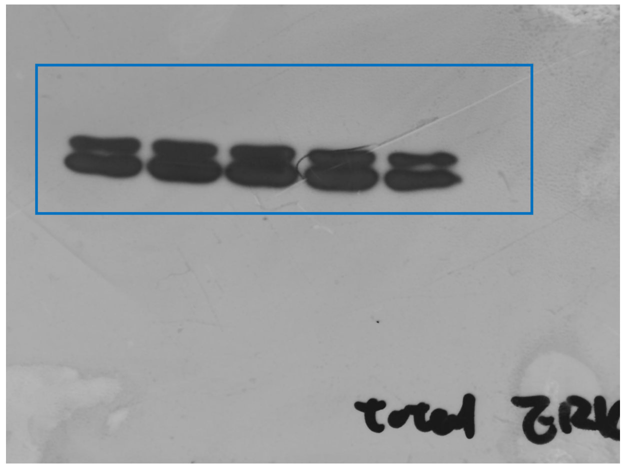

44 kDa
42 kDa

p-PKCζ

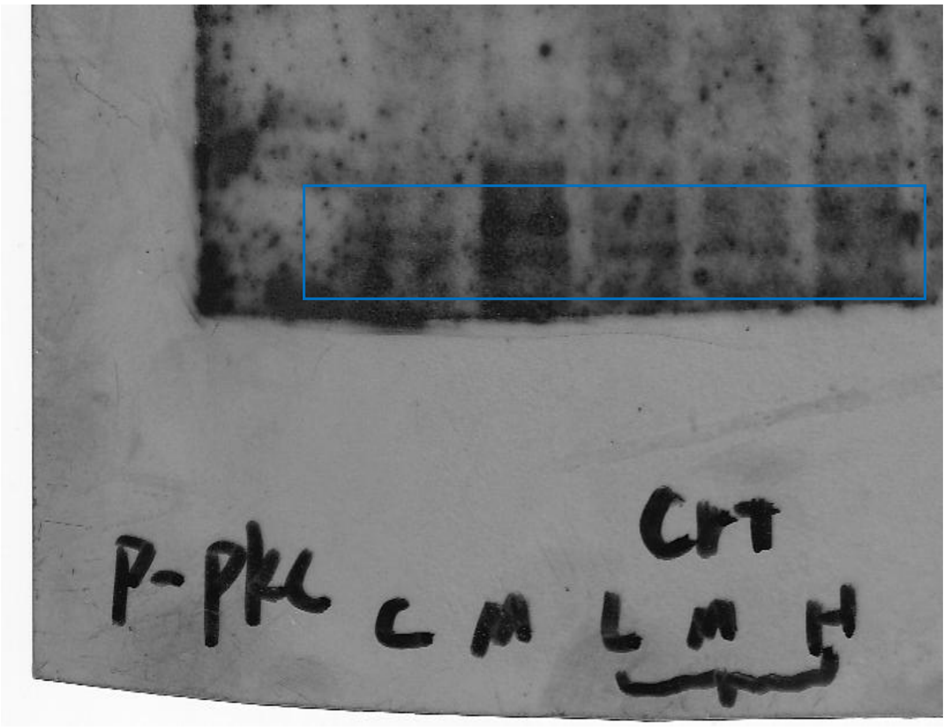

70 kDa

PKCζ

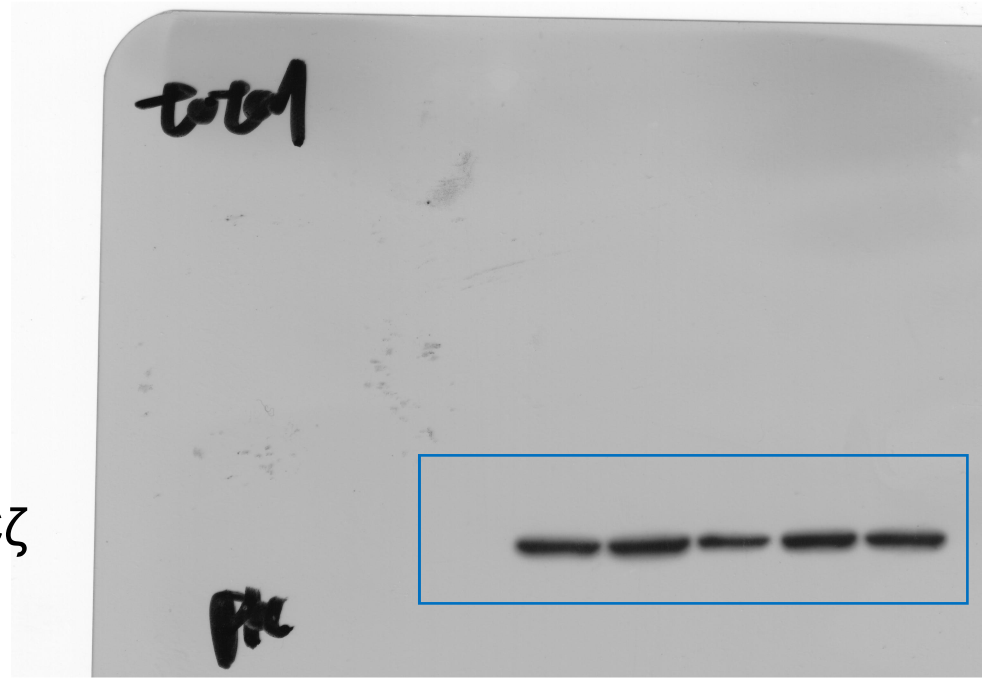

70 kDa

Fig. 7

A

Ctrl, MNNG. MNNG+crocetin (25, 50, 100 μM), MNNG+MG132

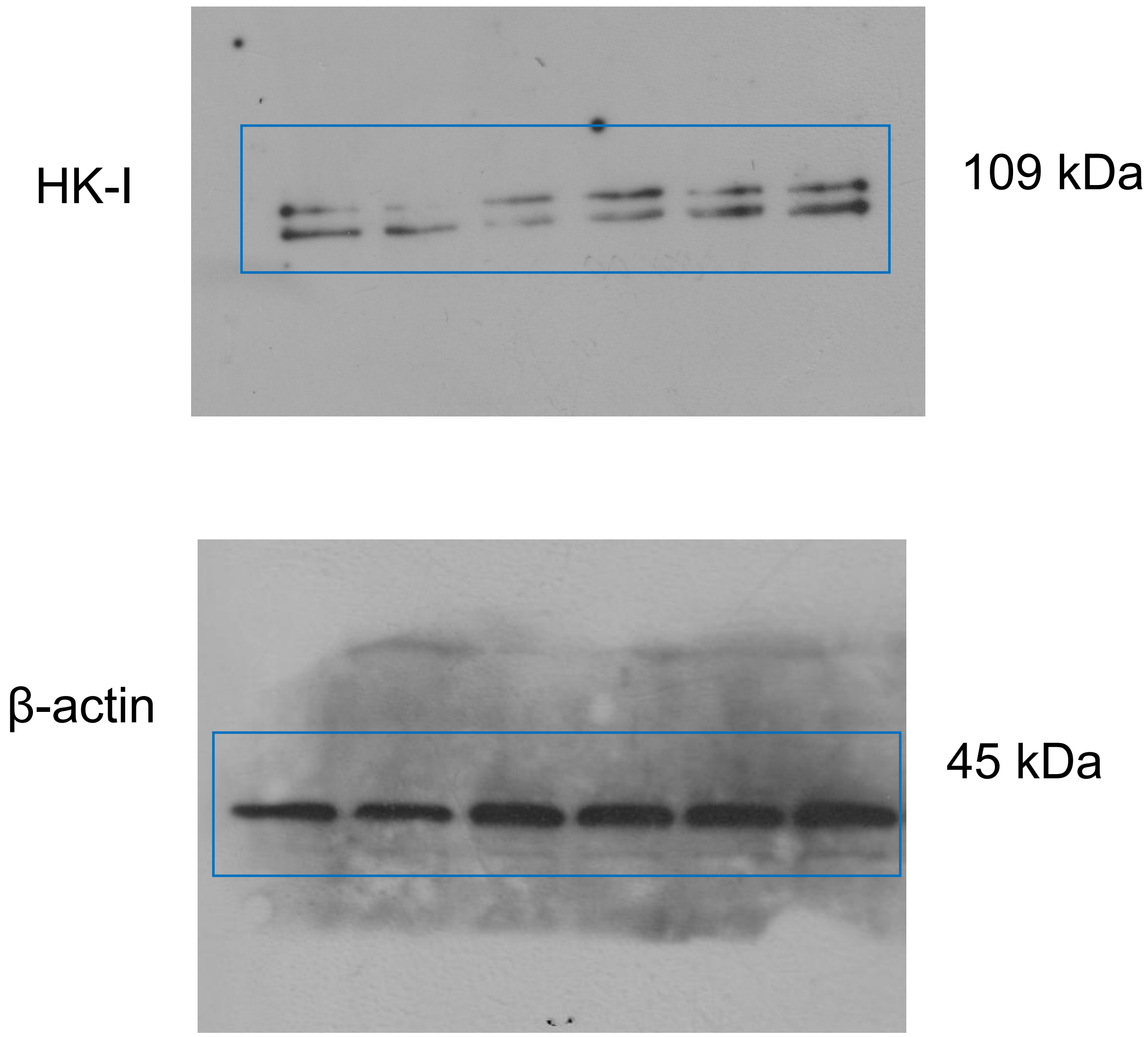

D

Ctrl, MNNG. MNNG+crocetin (25, 50, 100 μM)

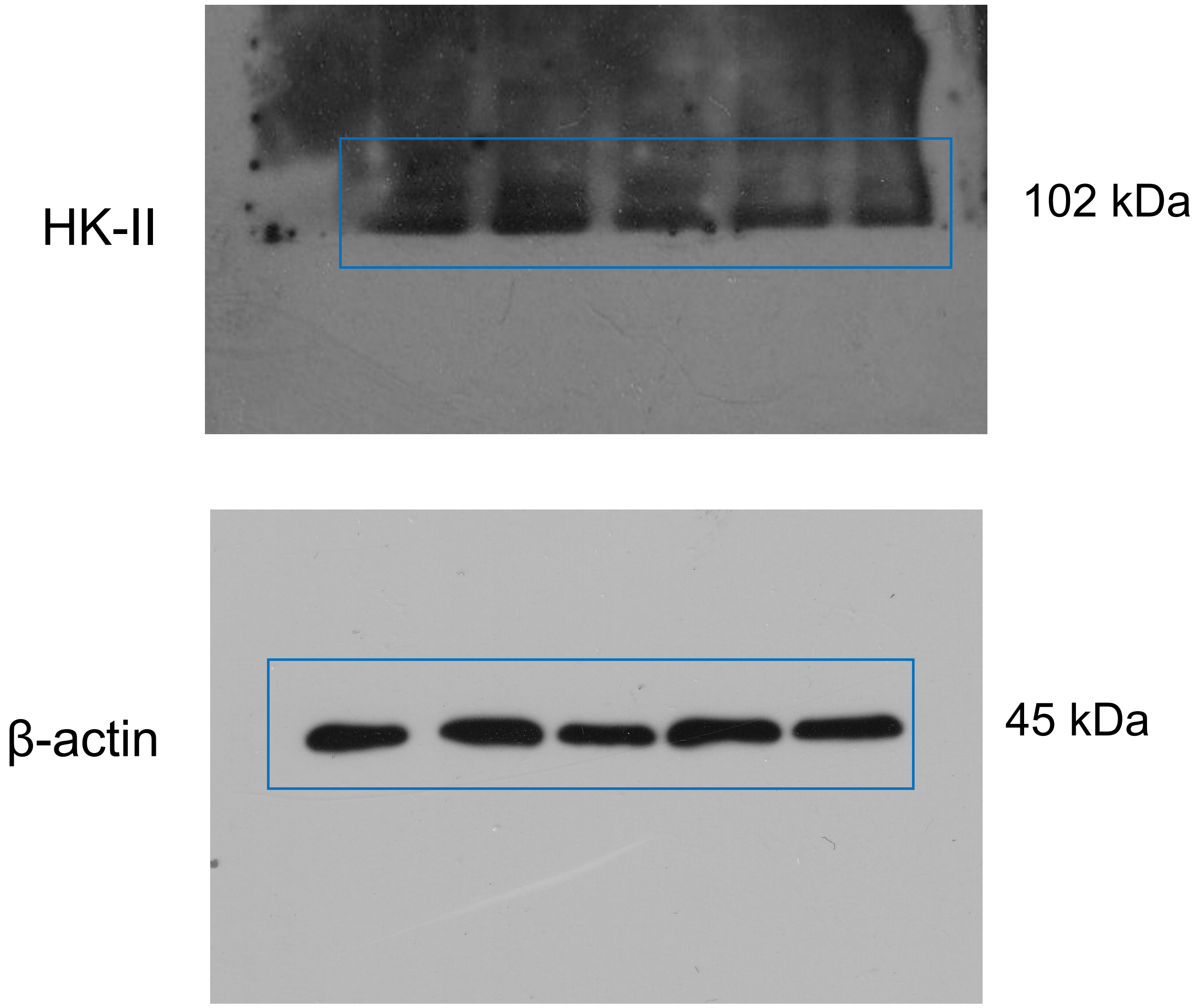

E Ctrl, MNNG, MNNG+crocetin (25, 50, 100 μM), IgG

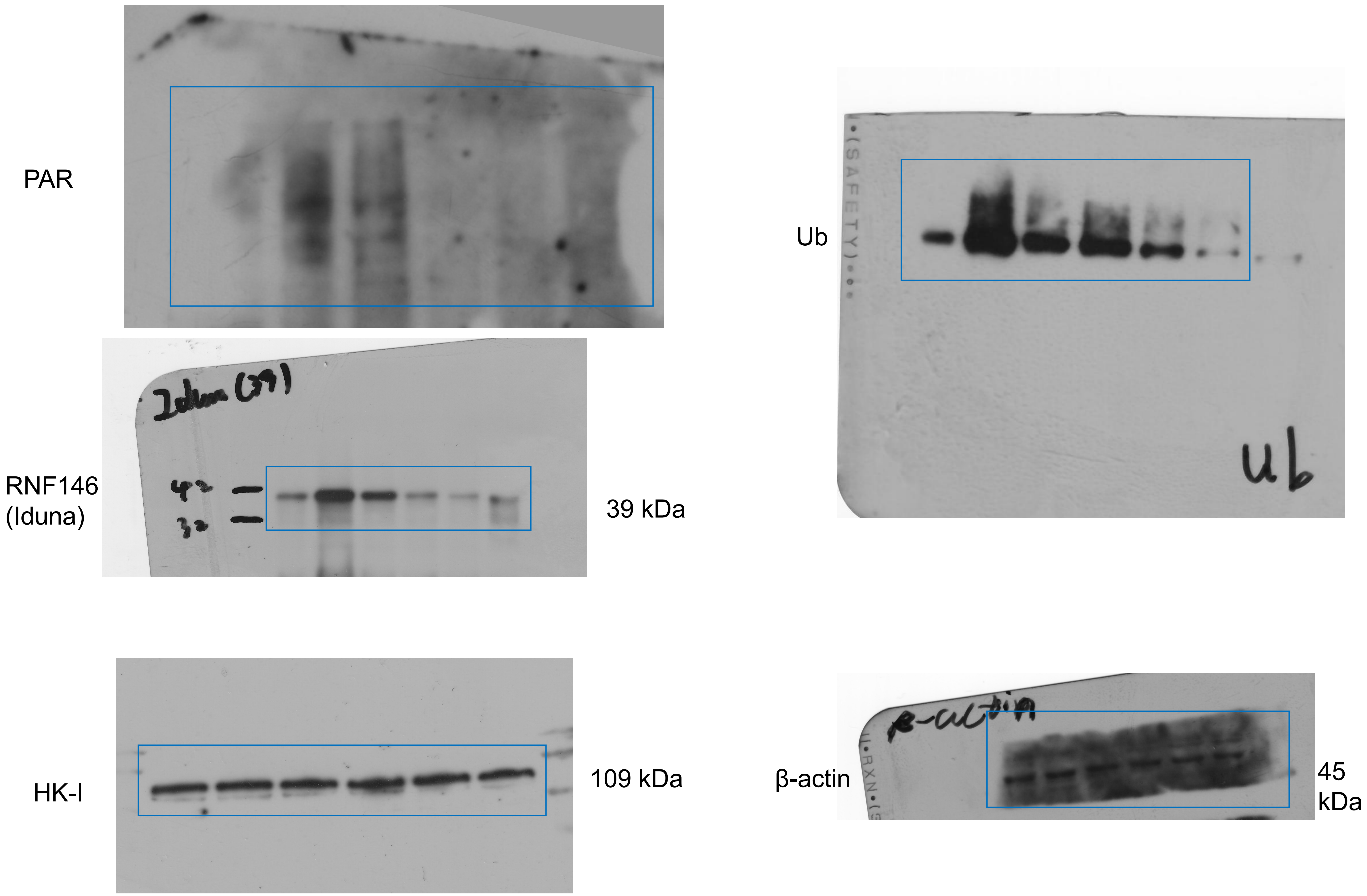

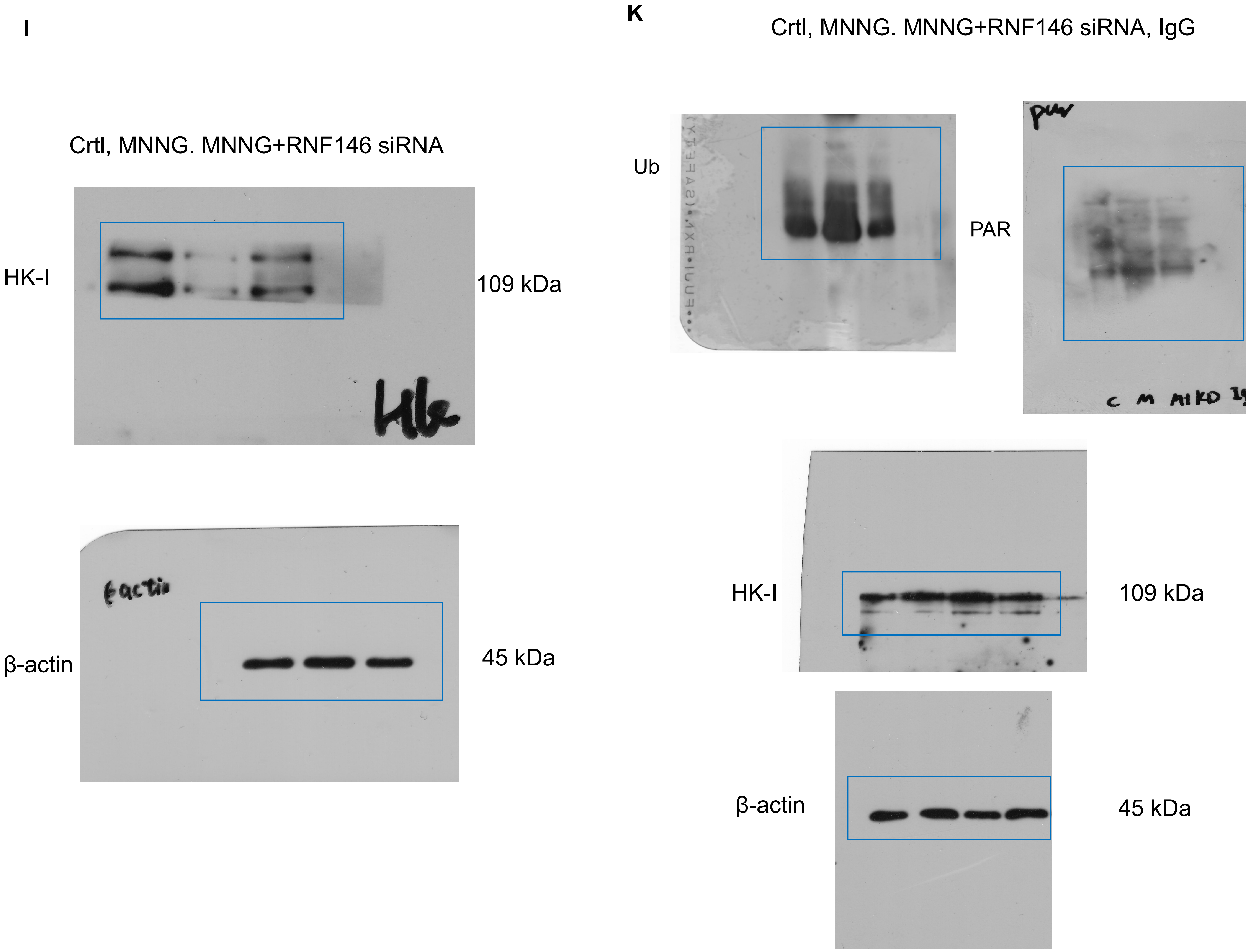

I
Crtl, MNNG. MNNG+RNF146 siRNA
HK-I
109 kDa
β-actin
45 kDa
K
Crtl, MNNG. MNNG+RNF146 siRNA, IgG
Ub
PAR
HK-I
109 kDa
β-actin
45 kDa

Fig. 8

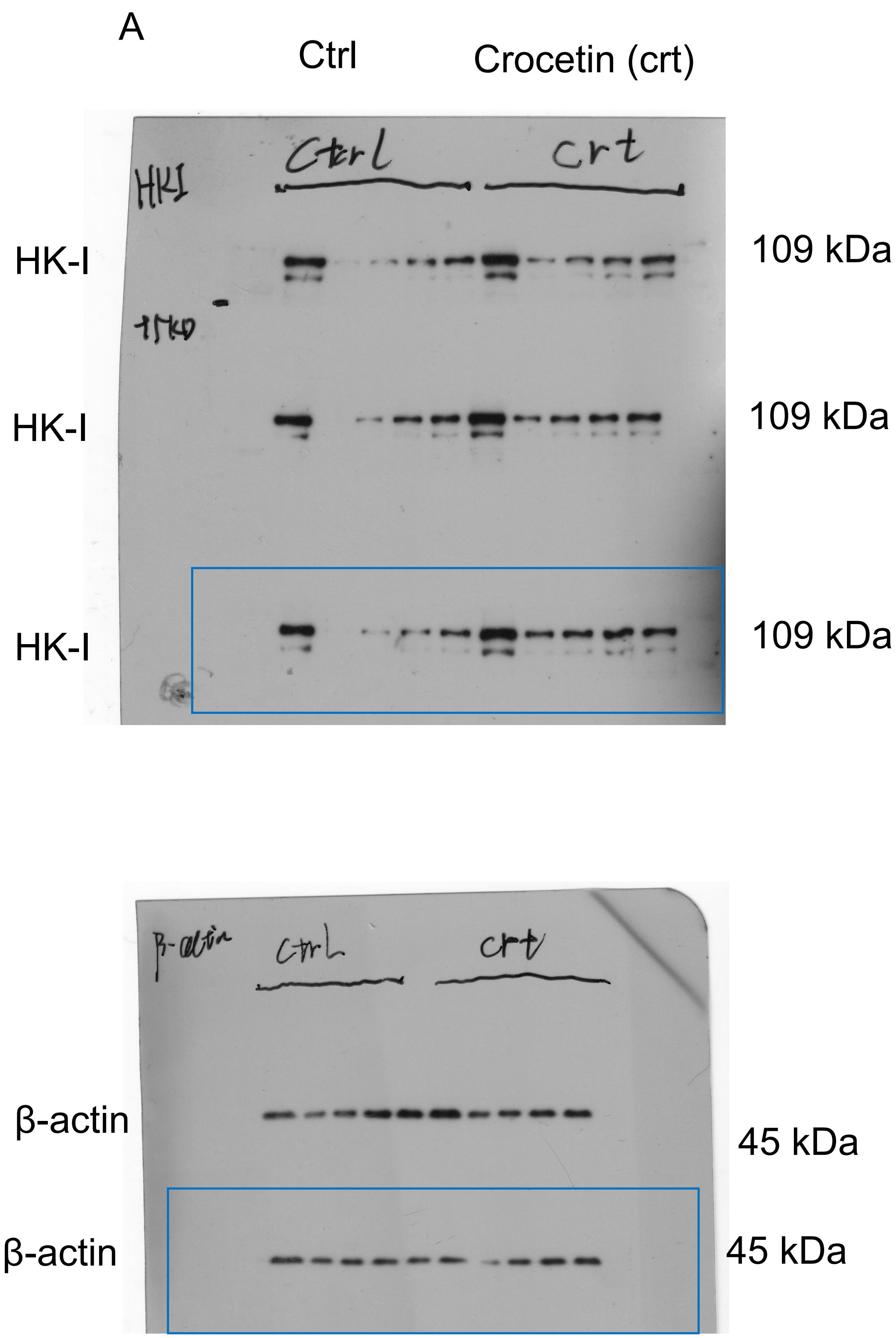
A
Ctrl
Crocetin (crt)
HK-I
109 kDa
HK-I
109 kDa
HK-I
109 kDa
β-actin
45 kDa
β-actin
45 kDa

Fig.S1

D Ctrl, OGD/R(4h). OGD/R(4h)+crocetin (5, 10, 25 μM), OGD/R(4h)+PJ34

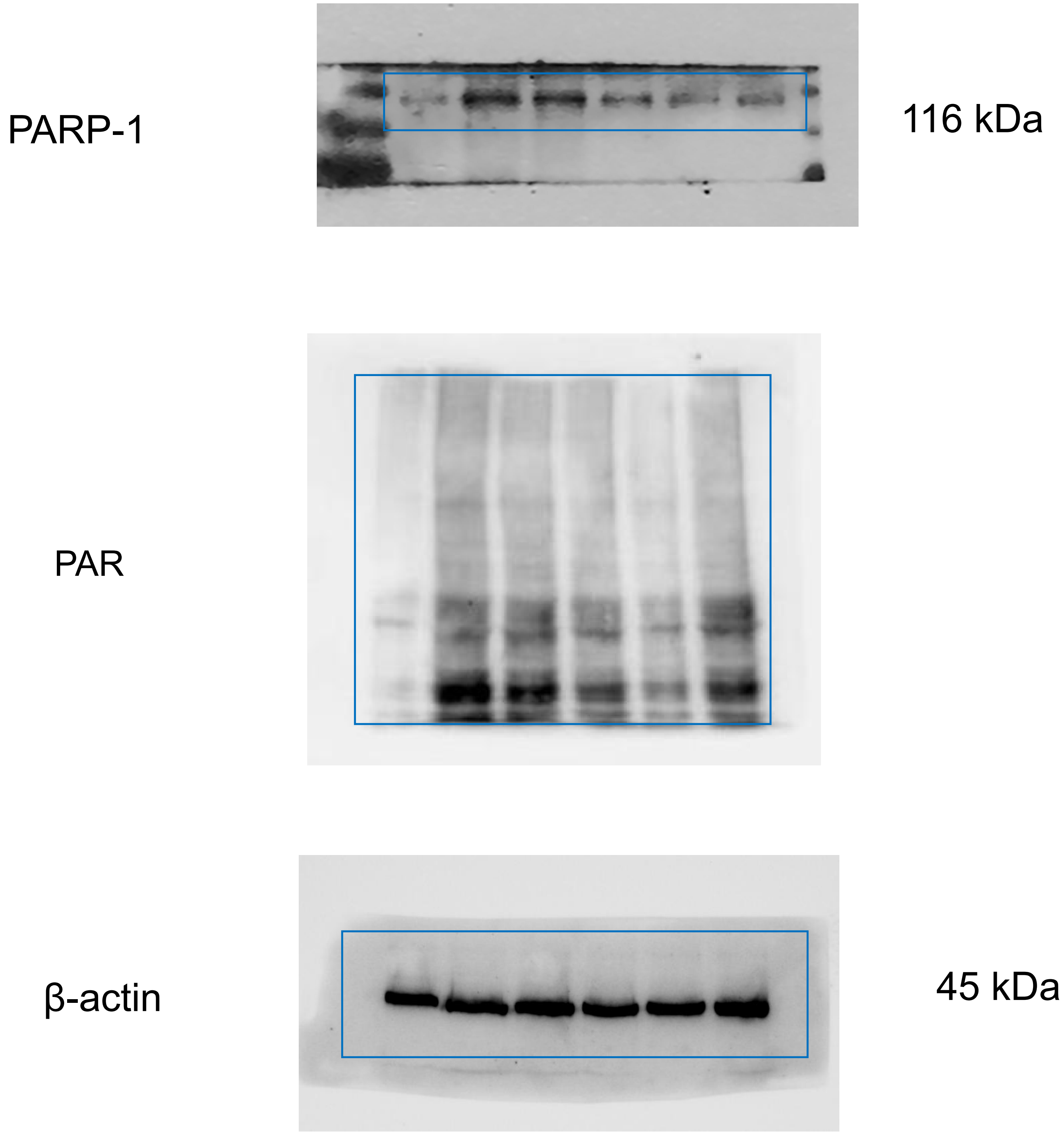

Fig.S1

L Ctrl, OGD/R(24h). OGD/R(24h)+crocetin (5, 10, 25 μM), OGD/R(24h)+PJ34

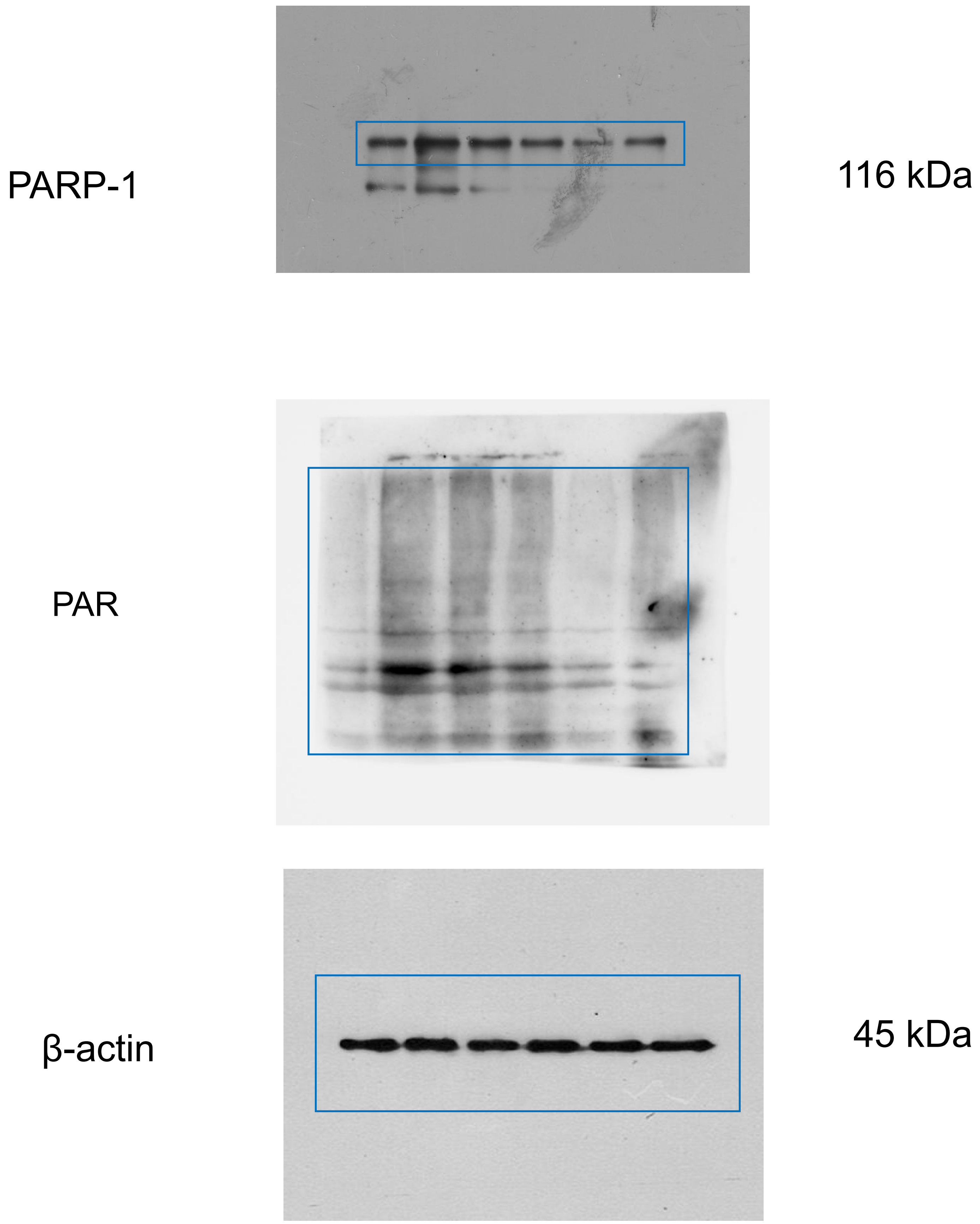

Fig.S4

Ctrl. MCAO, MCAO+crocin(20, 40 mg/kg).

A

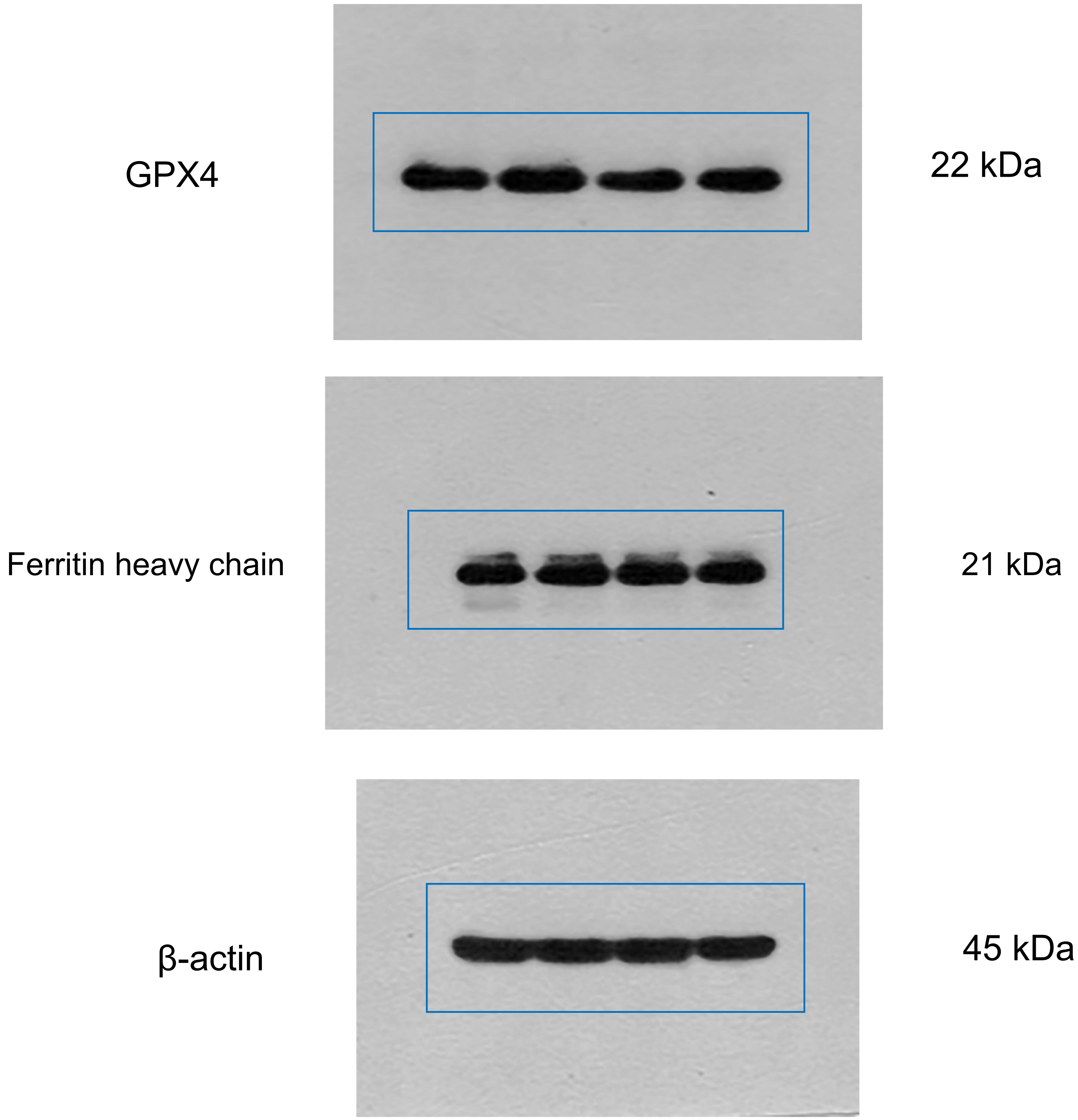

Fig.S6

A

RNF146 siRNA# (-, 1, 2, 3)

RNF146

39 kDa

β-actin

45 kDa

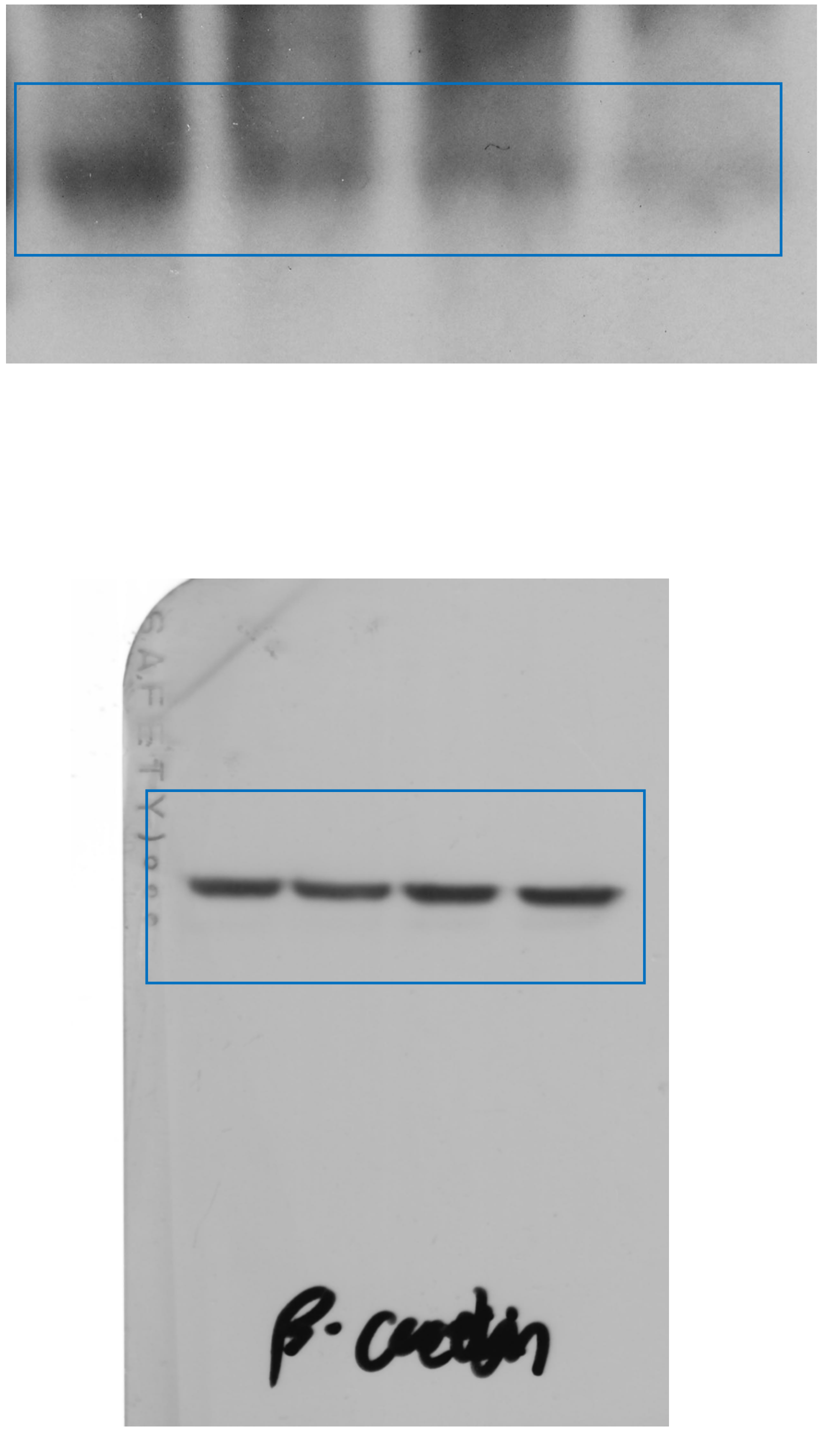

B

Crtl, MNNG. MNNG+NC siRNA

HK-I

109 kDa

β-actin

45 kDa

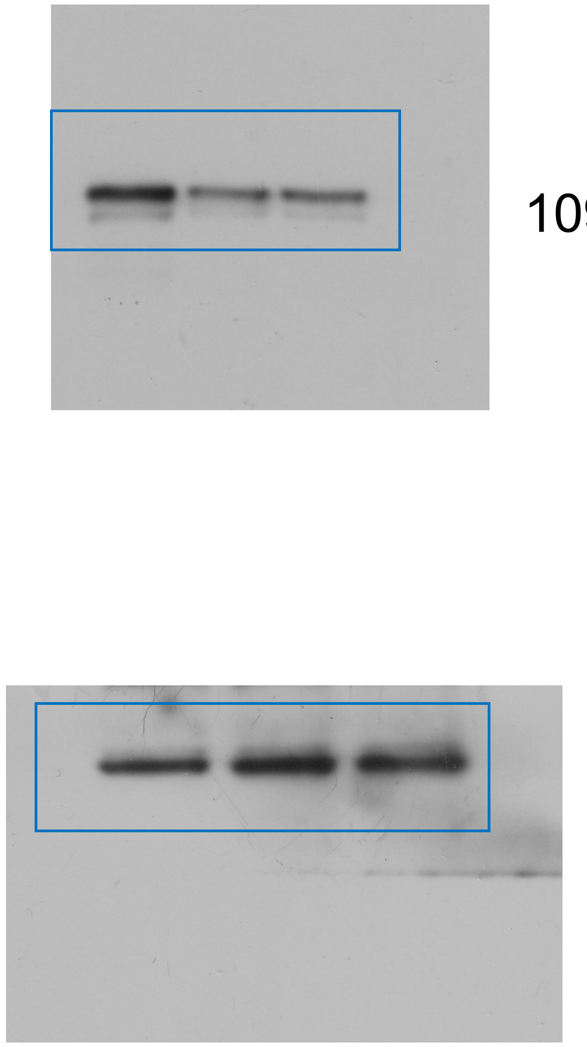

Fig. S7

B Ctrl. MCAO, MCAO+crocin(20, 40 mg/kg).

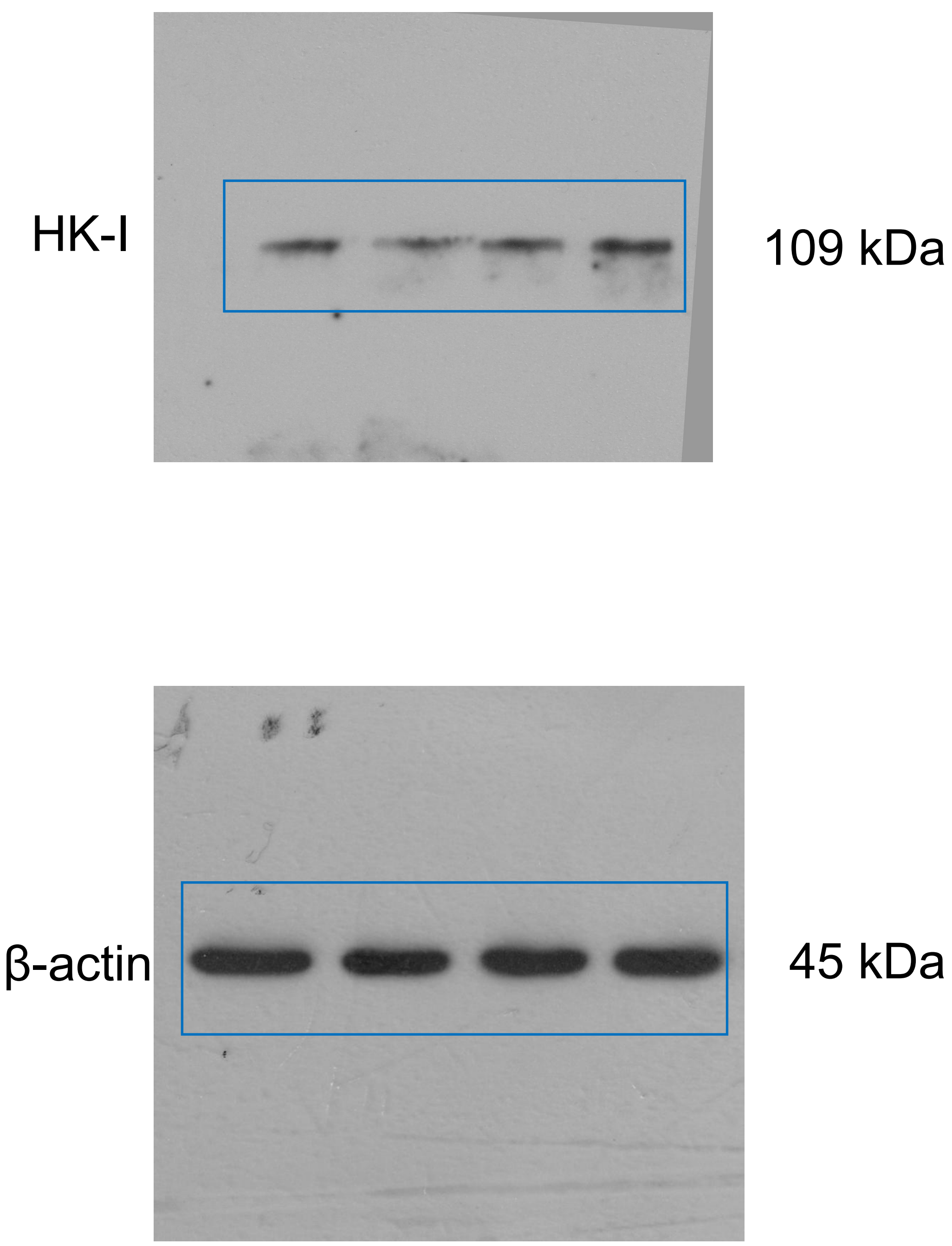

C

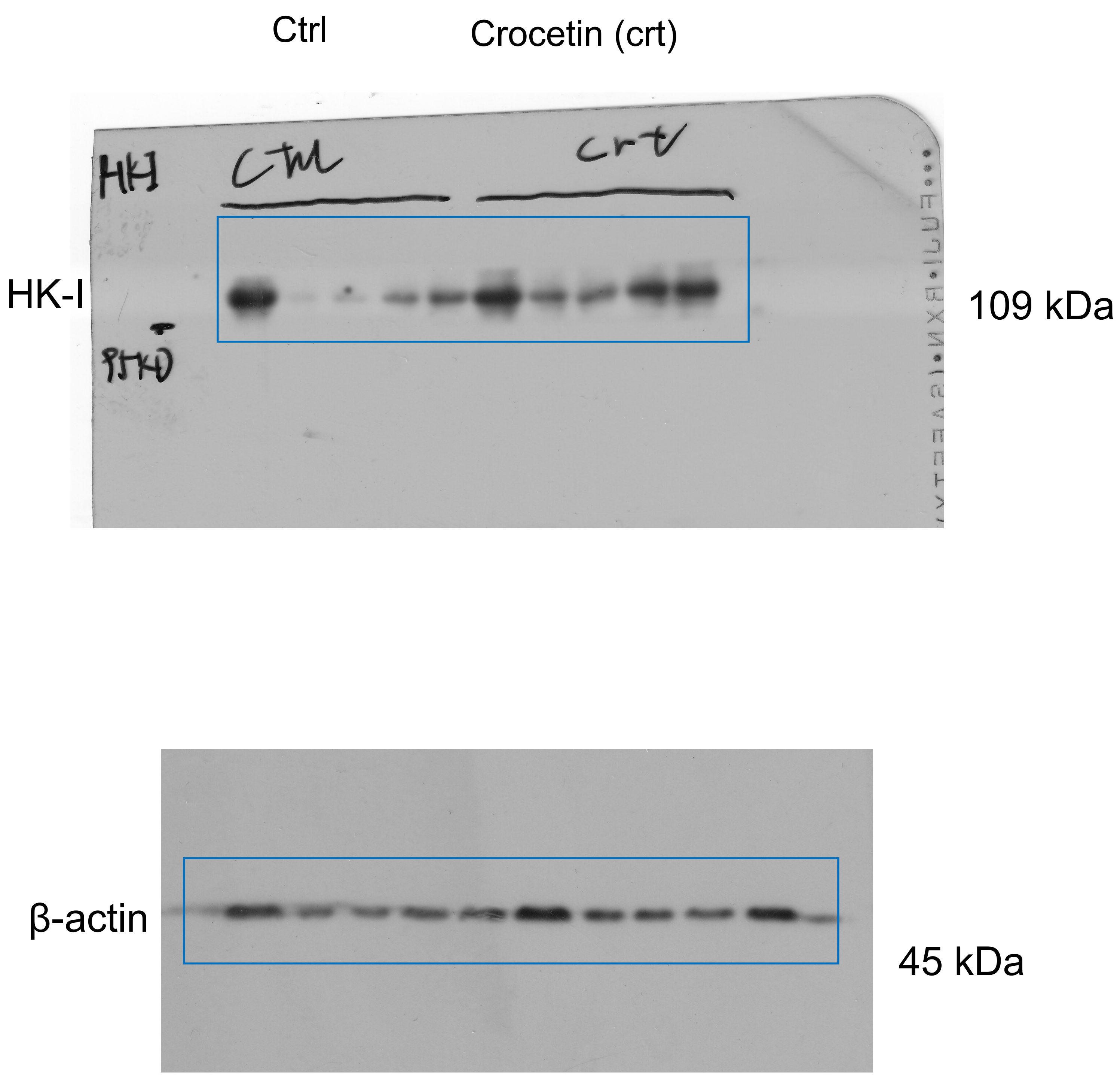

Supplement: Supplementary file 10 — Original Data File [file 41419_2023_5581_MOESM10_ESM.pdf]
